# Catalytic, Theoretical, and Biological Investigations of Ternary Metal (II) Complexes Derived from L-Valine-Based Schiff Bases and Heterocyclic Bases

**DOI:** 10.3390/molecules28072931

**Published:** 2023-03-24

**Authors:** Gopalakrishnan Sasikumar, Annadurai Subramani, Ramalingam Tamilarasan, Punniyamurthy Rajesh, Ponnusamy Sasikumar, Salim Albukhaty, Mustafa K. A. Mohammed, Subramani Karthikeyan, Zaidon T. Al-aqbi, Faris A. J. Al-Doghachi, Yun Hin Taufiq-Yap

**Affiliations:** 1 Department of Chemistry, St. Joseph’s College of Engineering, Chennai 600 119, Tamil Nadu, India; 2 Department of biochemistry, Dwaraka Doss Goverdhan Doss Vaishnav College, Chennai 600 106, Tamil Nadu, India; 3 Department of Chemistry, Vel Tech Multi Tech Dr. Rangarajan Dr. Sakunthala Engineering College, Chennai 600 062, Tamil Nadu, India; 4 Department of Physics, Vels Institute of Science, Technology and Advance Studies of Basic Science, Chennai 600 017, Tamil Nadu, India; 5 Department of Physics, Saveetha School of Engineering, SIMATS, Chennai 602 701, Tamil Nadu, India; 6 Department of Chemistry, College of Science, University of Misan, Maysan 62001, Misan, Iraq; 7 Radiological Techniques Department, Al-Mustaqbal University College, Hillah 51001, Babylon, Iraq; 8 Department of Physics, Periyar University Centre for Post Graduate and Research Studies, Dharmapuri 636 701, Tamil Nadu, India; 9 College of Agriculture, University of Misan, Al-Amara, Amarah 62001, Misan, Iraq; 10 Department of Chemistry, Faculty of Science, University of Basrah, Basra 61004, Basrah, Iraq; 11 Catalysis Science and Technology Research Centre, Faculty of Science, Universiti Putra Malaysia, Serdang 43400, Selangor, Malaysia; 12 Faculty of Science and Natural Resources, University Malaysia Sabah, Kota Kinabalu 88400, Sabah, Malaysia

**Keywords:** L-valine, ternary metal, binding interaction, Cu(II) complexes, antibacterial activity

## Abstract

A new series of ternary metal complexes, including Co(II), Ni(II), Cu(II), and Zn(II), were synthesized and characterized by elemental analysis and diverse spectroscopic methods. The complexes were synthesized from respective metal salts with Schiff’s-base-containing amino acids, salicylaldehyde derivatives, and heterocyclic bases. The amino acids containing Schiff bases showed promising pharmacological properties upon complexation. Based on satisfactory elemental analyses and various spectroscopic techniques, these complexes revealed a distorted, square pyramidal geometry around metal ions. The molecular structures of the complexes were optimized by DFT calculations. Quantum calculations were performed with the density functional method for which the LACVP++ basis set was used to find the optimized molecular structure of the complexes. The metal complexes were subjected to an electrochemical investigation to determine the redox behavior and oxidation state of the metal ions. Furthermore, all complexes were utilized for catalytic assets of a multi-component Mannich reaction for the preparation of -amino carbonyl derivatives. The synthesized complexes were tested to determine their antibacterial activity against *E. coli*, *K. pneumoniae*, and *S. aureus* bacteria. To evaluate the cytotoxic effects of the Cu(II) complexes, lung cancer (A549), cervical cancer (HeLa), and breast cancer (MCF-7) cells compared to normal cells, cell lines such as human dermal fibroblasts (HDF) were used. Further, the docking study parameters were supported, for which it was observed that the metal complexes could be effective in anticancer applications.

## 1. Introduction

The discovery of cisplatin (*cis*-dichlorodiammine platinum(II)) and its subsequent use as a drug for the treatment of numerous human tumors sparked the development of advanced medicinal inorganic chemistry, which, in turn, was facilitated by inorganic chemists’ extensive knowledge of the coordination and redox characteristics of metal ions [1,2]. Schiff bases [3,4] have been used in countless organic syntheses and analyses of biomedical activities, such as anticancer [3], anti-inflammatory [4], anti-tubercular [5], antioxidant [6], and antibacterial [7], due to the occurrence of a bonding interaction with core metal ions upon complexation. The presence of azomethane bonds has demonstrated a good ability to coordinate metal ions, thereby yielding metal complexes with interesting structural and electronic properties. Additionally, it has been noted that a variety of processes have benefited from the mixed ligand complexes of amino-acid-based Schiff bases and heterocyclic bases with transition metal ions [8]. These processes have improved their functionality and product selectivity. More efficient methods of synthesis and the thermal stability of these complexes have greatly contributed to their potential biological uses as metal complexes in applications involving antibacterial activity and in vitro cytotoxicity. Significant efforts have been made in the field of catalysis to achieve Mannich reactions in water using various Lewis or Bronsted acid catalysts, such as Bi(OTf) 3.4H2O [9], scandium tris(dodecyl sulfate) [10], HBF4 [11], dodecylbenzenesulfonic acid [12], acidic liquids [13], SO3H-functionalized liquids [14], and amino acids [15]. This study aims to present conventional and sustainable ways of creating and utilizing metal (II) complex salts as catalysts. We report the synthesis of Cu(II), Ni(II), Co(II), and Zn(II) complexes using Schiff bases (L-valine with 5-bromosalicylaldehyde) and heterocyclic bases (1,10-phenanthroline or 2,2′-bipyridyl). To further comprehend the structural properties of these complexes, a wide range of physical techniques were used for their characterization. Herein, Schiff base metal complexes were used as antibacterial agents against bacterial cells at various concentrations. Similarly, in the quest for obtaining more effective and selective antitumor agents, this study investigated the cytotoxic effects of the developed Cu(II) complexes on three distinct human cancer cell lines. Nontumorigenic human dermal fibroblast cells were used as a normal cell line to test the toxicity of the complexes. Consequently, theoretical research was conducted on all metal(II) complexes to examine their orbitals. In addition, molecular docking investigations were used to determine how well the complexes bind to the active site of human thymidylate synthase. Therefore, through the abovementioned contributions, we report Co(II), Ni(II), Cu(II), and Zn(II) complexes containing a tridentate-based potassium (E)-2-((5-bromo-2-hydroxy benzylidene)amino)-3-methylbutanoate (HL) ligand, which also supports some other coligands. Furthermore, the molecular structures of the complexes were optimized by DFT calculations. The synthesized metal complexes have been utilized for catalytic and biological applications.

## 2. Materials and Methods

### 2.1. Experimental Section

#### 2.1.1. Reagents and Characterization

The chemical compound 5-bromosalicylaldehyde was obtained from Alfa Aesar, and the AnalaR-grade products of L-valine, metal(II) salts, 1,10-phenanthroline, and 2,2′-bipyridine were obtained from Sisco Research Laboratories. The solvents utilized for synthesis were dried by a traditional procedure [16]. A Perkin-Elmer 2400 elemental analyzer was used to evaluate the complexes’ CHN. PerkinElmer FT-IR spectroscopy was used to acquire FT-IR spectra at 4000 and 400 cm^−1^, and UV–Vis patterns of samples were studied (Perkin-Elmer). X-ray diffraction patterns were acquired using BRUKER D8 with CuK_α_ source. Cyclic voltammograms were acquired at room temperature under N_2_ atmosphere using a CHI600A electrochemical analyzer. At room temperature, EPR spectra were obtained using a JES-X310 EPR spectrometer.

#### 2.1.2. Preparation of Potassium (E)-2-((5-bromo-2-hydroxy benzylidene)amino)-3-methylbutanoate (HL)

An L-valine solution in methanol (0.1 mol in 50 mL) was refluxed with KOH at 50 °C for 60 min. The filtered, transparent solution was then refluxed with a methanolic solution of 5-bromosalicylaldehyde (0.1 mol in 50 mL). This process was completed after one hour of stirring at 60 °C. Subsequently, the mixture was crystallized by adding an excessive concentration of diethyl ether and then recrystallized, filtered, and dried. Yield: (80%). Color: yellow. M.p.110 °C. Anal. Calc for C_12_H_13_BrKNO_3_: found (calc.) (%): C, 48.56(48.18); H, 4.12(4.32); N, 4.38(4.68); ^1^H NMR (DMSO-d_6,_δ, PPM) 9.88(s, 1H, Ar-OH), 8.24 (s, 1H, N=CH), 7.54 (s, 1H, Ar-H), 6.54–7.37 (m, 1H, Ar-H), 3.62–3.68 (q,1H, valine C-H, 0.99 Hz), and 1.35–1.37 (m, 1H, valine C-H, 9.00 Hz). (d,3H,valine CH-(CH_3_)_2_, 2.99 Hz). IR (ν, cm^−1^): 3380 (OH); 1643 (C=N); 1594 (COO^−^); 1405 (COO); (λ_max_/nm (ε/M^−1^cm^−1^dm^3^) in MeOH: 409(500), 353(180), and 264(1590).

### 2.2. Preparation of Complexes

#### 2.2.1. Preparation of [Co(L)(phen)] (**1a**)

A heated methanolic liquid of potassium (E)-2-((5-bromo-2-hydroxy benzylidene)amino)-3-methylbutanoate (HL) (0.1 mol) was progressively introduced with steady mixing of CoCl_2_.6H_2_0 (0.1 mol), and the solution was refluxed for 3 h. Then, the solution was refluxed for three hours with 1,10 phenanthroline (0.1 mol). After cooling to ambient temperature, the precipitate was collected, filtered, and repeatedly washed with methanol. Yield: 0.49 g (80%). Color: brown. M.p. > 300 °C (dec.). Anal. Calc for C_24_H_20_BrCoN_3_O_3_: found (calc.) (%): C, 53.63(53.65); H, 3.72(3.75); N, 7.81(7.82); selected IR data (ν, cm^−1^): 1636 (C=N), 1587 (COO), 1341(COO), 549 (M-O), and 457 (M-N). UV–Vis in MeOH [*λ*_max_/nm (ε/M^−1^cm^−1^)]: 228(33,895), 269(25,834), 390(1209), 477(152), and 615(58). ESI-MS (*m/z*): [Co(L)(phen)]^+^ (537.08).

#### 2.2.2. Preparation of [Ni(L)(phen)] (**1b**)

Complexes **1b**–**1d** were prepared using the same method introduced for complex-**1a**. Complex-**1b** from NiCl_2_.6H_2_O (0.1 mole). Yield: 0.47 g (80%). Color: pale green. M.p. > 300 °C (dec.). Anal. Calc for C_24_H_20_BrN_3_NiO_3_: found (calc.) (%): C, 53.66(53.68); H, 3.73(3.75); N, 7.80(7.82); selected IR data (ν, cm^−1^): 1646 (C=N), 1590 (COO), 1325 (COO), 541 (M-O), and 454 (M-N). UV–Vis in MeOH [*λ*_max_/nm (ε/M^−1^cm^−1^)]: 254(57302), 288(36331), 389(3657), and 623(5.3). ESI-MS (*m/z*): [Ni(L)(phen)]^+^ (536.92).

#### 2.2.3. Preparation of [Cu(L)(phen)] (**1c**)

Complex-**1c** from CuCl_2_.2H_2_O (0.1 mole). Yield: 0.56 g (88%). Color: dark green. M.p. > 300 °C (dec.). Anal. Calc for C_24_H_20_BrCuN_3_O_3_: found (calc.) (%): C, 53.19 (53.20); H, 3.70(3.72); N, 7.72(7.75); selected IR data (ν, cm^−1^): 1627 (C=N), 1592 (COO), 1325 (COO), 554 (M-O), and 455 (M-N). UV–Vis in MeOH [*λ*_max_/nm (ε/M^−1^cm^−1^)]: 259(86,422), 284(58,665), 372(1502), and 637(17). ESI-MS (*m/z*): [Cu(L)(phen)]^+^ (541.78).

#### 2.2.4. Preparation of [Zn(L)(phen)] (**1d**)

Complex-**1d** from Zn(CH_3_COOH)_2_.2H_2_O (0.1 mole). Yield: 0.42 g (67%). Color: Pale yellow. M.p. > 300 °C (dec.). Anal. Calc for C_24_H_20_BrN_3_O_3_Zn: found (calc.) (%): C, 52.98(53.02); H, 3.69(3.71); N, 7.72(7.73); selected IR data (ν, cm^−1^): 1622 (C=N), 1574 (COO), 1343 (COO), 549 (M-O), and 510 (M-N). UV–Vis in MeOH [*λ*_max_/nm (ε/M^−1^cm^−1^)]: 270(35,064), 292(9154), and 373(4019)). ESI-MS (*m/z*): [Zn(L)(phen)]^+^ (543.28).

#### 2.2.5. Preparation of [Co(L)(bpy)] (**1e**)

A similar procedure used to create complex 1a was utilized to develop complexes **1e**–**1h**, but instead of 1,10–phenanthroline, 0.1 mole of 2,2′–bipyridyl was used. Complex-**1e** from CoCl_2_.6H_2_0 (0.1 mole.). Yield: 0.48 g (84%). Color: Brown. M.p. > 300 °C (dec.). Anal. Calc for C_22_H_20_BrCoN_3_O_3_: found (calc.) (%): C, 51.46(51.48); H, 3.92(3.93); N, 8.18(8.19); selected IR data (ν, cm^−1^): 1623 (C=N), 1592 (COO), 1368 (COO), 562 (M-O), and 488 (MνN). UV–Vis in MeOH [*λ*_max_/nm (ε/M^−1^cm^−1^)]: 249(19,579), 301(8048), 389(854), 463(127), and 619(65). ESI-MS (*m/z*): [Co(L)(bpy)]^+^ (513.03).

#### 2.2.6. Preparation of [Ni(L)(bpy)] (**1f**)

Complex-**1f** from NiCl_2_.6H_2_O (0.1 mole). Yield: 0.46 g (79%). Color: Pale green. M.p. > 300 °C (dec.); Anal. Calc for C_22_H_20_BrN_3_NiO_3_: found (calc.) (%): C, 51.49(51.51); H, 3.91(3.93); N, 8.17(8.19); selected IR data (ν, cm^−1^): 1647 (C=N), 1591 (COO), 1326 (COO), 542 (M-O), and 503 (M-N). UV–Vis in MeOH [*λ*_max_/nm (ε/M^−1^cm^−1^)]: 238(4056), 298(2094), 379(920), and 625(3.4). ESI-MS (*m/z*): [Ni(L)(bpy)]^+^ (512.89).

#### 2.2.7. Preparation of [Cu(L)(bpy)] (**1g**)

Complex-**1g** from CuCl_2_.2H_2_O (0.1 mole). Yield: 0.52 g (83%). Color: Green. M.p. > 300 °C (dec.). Anal. Calc for C_22_H_20_BrCuN_3_O_3_: found (calc.) (%): C, 51.00(51.02); H, 3.88(3.89); N, 8.09(8.11); selected IR data (ν, cm^−1^): 1627 (C=N), 1593(COO), 1325 (COO), 551(M-O), and 503 (M-N). UV–Vis in MeOH [*λ*_max_/nm (ε/M^−1^cm^−1^)]: 240(13846), 300(11085), 375(144), and 642(24). ESI-MS (*m/z*): [Cu(L)(bpy)]^+^ (517.69).

#### 2.2.8. Preparation of [Zn(L)(bpy)] (**1h**)

Complex-**1h** from Zn(CH_3_COOH)_2_.2H_2_O (0.1 mole). Yield: 0.41 g (67%). Color: Pale yellow. M.p. > 300 °C (dec.). Anal. Calc for C_22_H_20_BrN_3_O_3_Zn: found (calc.) (%): C, 50.81(50.84); H, 3.86(3.88); N, 8.06(8.09); selected IR data (ν,cm^−1^): 1635 (C=N), 1595 (COO), 1314 (COO), 548 (M-O), and 505 (M-N).UV–Vis in MeOH [*λ*_max_/nm (ε/M^−1^cm^−1^)]: 274(20198), 294(16748), and 374(8425). ESI-MS (*m/z*): [Zn(L)(bpy)]^+^ (519.38).

### 2.3. In Vitro MTT Assay

According to the previous literature [17,18], the cytotoxic activity of compounds [Cu(L)(phen)] **1c** and [Cu(L)(bpy)] **1g** was assessed with respect to three tumor cell lines, including the A549, HeLa, and MCF-7 lines, as well as NHDF normal cells. The medium was switched to DMEM with 1% FBS after one day of growth in Dulbecco’s Modified Eagle Medium with 10% FBS. After one day, 2, 5, 10, 25, 50, and 100 µL doses of the produced compounds diluted in DMSO were applied to the cells, which were then incubated. Each well was then filled with 10 µL of MTT (5 mg/mL) and maintained in this state for 3 h. Farmazone crystals were produced and dispersed in 100 µL of DMSO; then, the absorbance at 570 nm was calculated using an ELISA spectrometer. The proportion of viable cells was determined using the following formula [17,19,20]:

Cell viability (%) = (*A*_570_ nm of treated samples/*A*_570_ nm of control samples) × 100

### 2.4. Molecular Modeling

Complexes were modeled molecularly by Maestro software coupled with the Schrodinger equation. The complex **1**(**a**–**h**) 3D structures were first sketched in the maestro builder panel before being optimized in the Ligprep system. The complexes’ tailored structures were suitable for docking with receptors. The receptor thymidylate synthase (PDB ID: 1HZW) was chosen from the protein data bank (http://www.rcsb.org, accessed on 22 October 2022). Utilizing the force field OPLS-2005, the receptor was also extensively tuned in the protein preparation wizard. The grid parameters were generated by supplying coordinates of −23.213, 43.011, and 21.67 Å and maintaining a diameter of 20 × 20 × 20 Å. Finally, docking programs were run in the maestro workspace for optimized complexes with receptor grids and binding interactions (3D and 2D).

### 2.5. Computational Analysis

Jaguar software, which is included in the Schrödinger suite 2017, was used to run the computational program employed in this study. Density functional theory (DFT) and the B3LYP/LACVP++ basis set were used to examine the optimal geometries and perform molecular orbital analyses (HOMO–LUMO) of the complexes.

### 2.6. The General Process for the Synthesis of β-Amino Carbonyl Derivative

Simple/substituted aryl aldehyde (9.423 × 10^−3^ mmol; 1.0 equiv.) is mixed with aniline (9.894 × 10^−3^ mmol; 1.05 equiv.) and cyclohexanone (1.038 × 10^−2^ mmol; 1.05 equiv.), and optimized catalyst concentration of substitued2,2-bipyridyl/phenanthroline metal (II) salts **1**(**a**–**h**) (5.44 × 10^−2^ mmol) is added in the presence of 40 mL of dry CH_3_CN for 5–125 min under refluxing conditions to yield substituted β-Amino carbonyl derivatives **2**(**a**–**e**) at 55–98% after purification. All the synthesized *β*-Amino carbonyl derivatives are thoroughly analyzed via spectral and analytical tools and verified through an examination of the literature.

## 3. Results and Discussion

### 3.1. Results and Discussion

**Scheme 1:**
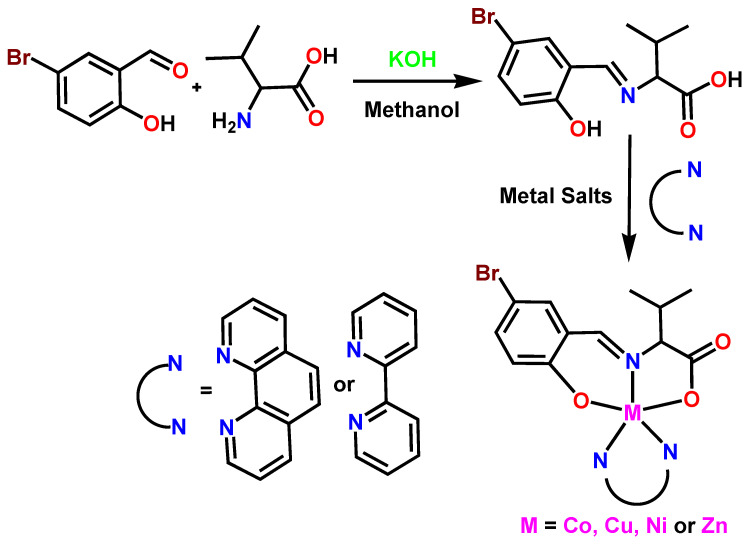
Synthetic route of Co(II), Ni(II), Cu(II), and Zn(II) complexes with tridentate ligand (HL) and bidentate diamines (1,10-phenanthroline or 2,2′-bipyridyl).

The metal(II) complexes **1**(**a**–**h**) were synthesized by a stoichiometric (1:1:1) template reaction of a methanolic solution containing ligand potassium (E)-2-((5-bromo-2-hydroxybenzylidene) amino)-3-methylbutanoate (HL), heterocyclic bases (1,10-phenanthroline or 2,2′-bipyridyl), and hydrated metal(II) salts (Scheme 1). Table 1 depicts the physical and elemental analyses of the metal(II) complexes. The complexes presented high yields and stability at ambient temperatures. The complexes were highly soluble in many solvents, such as methanol, acetonitrile, DMSO, and DMF, and were partially soluble in water.

### 3.2. Spectral Characterization

#### 3.2.1. FT-IR Spectral Analysis

As shown in Table 2, the FT-IR spectra of the mixed ligand complexes **1**(**a**–**h**) were measured in the 450–4000 cm^−1^ wavelength range (Figure S1 from Supplementary Materials). A broad peak was observed in the ligand 3300–3500 cm^−1^ region due to the -OH stretching vibration of the ligand (HL), which was not present in the complexes for which the coordination mode of the deprotonated hydroxyl group with metal ions was confirmed. The C=N- (azomethine) stretching frequency of the ligand showed a strong absorbance peak at 1643 cm^−1^; a shift toward the higher wavelength region (1610–1635 cm^−1^) of the metal(II) complexes revealed that the azomethine nitrogen had coordinated with metal ions (Table 2) [21]. The bands at (1570–1590 cm^−1^) and (1350–1380 cm^−1^) were attributed to the asymmetric and symmetric vibrational frequencies, respectively, of the carboxyl groups present in the amino acid [22,23,24,25]. The absence of a band in the 1700–1750 cm^−1^ region suggested that the COO^−^ group of L-valine was coordinated with the central metal ion and that the difference in the frequency value between the asymmetric and symmetric stretching vibrations of the complexes lay between 180 and 216 cm^−1^, for which the latter is practically larger than that of a free carboxylate ion [26]. These findings supported the notion of a monodentate coordination of the carboxyl group of L-valine with metal ions. The spectra of all the complexes showed a strong band at 725–727 cm^−1^, thus indicating that –CH was out of the plane in the center ring of 1,10-phenanthroline. The lower frequency regions of the metal(II) complexes around 540–565 cm^−1^ and 450–505 cm^−1^, which are attributed to the (M-O) and (M-N) bands, respectively, confirmed the coordination of ligands with metal ions [27,28]. Hence, all the above facts are in good agreement with the complexes coordinated as a tridentate mode.

#### 3.2.2. UV–Visible Spectral Analysis

**Figure 1:**
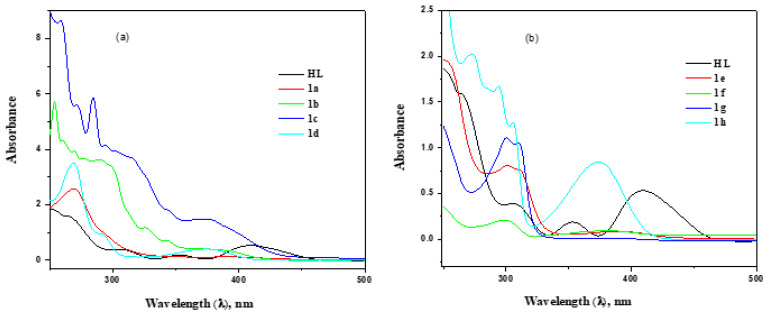

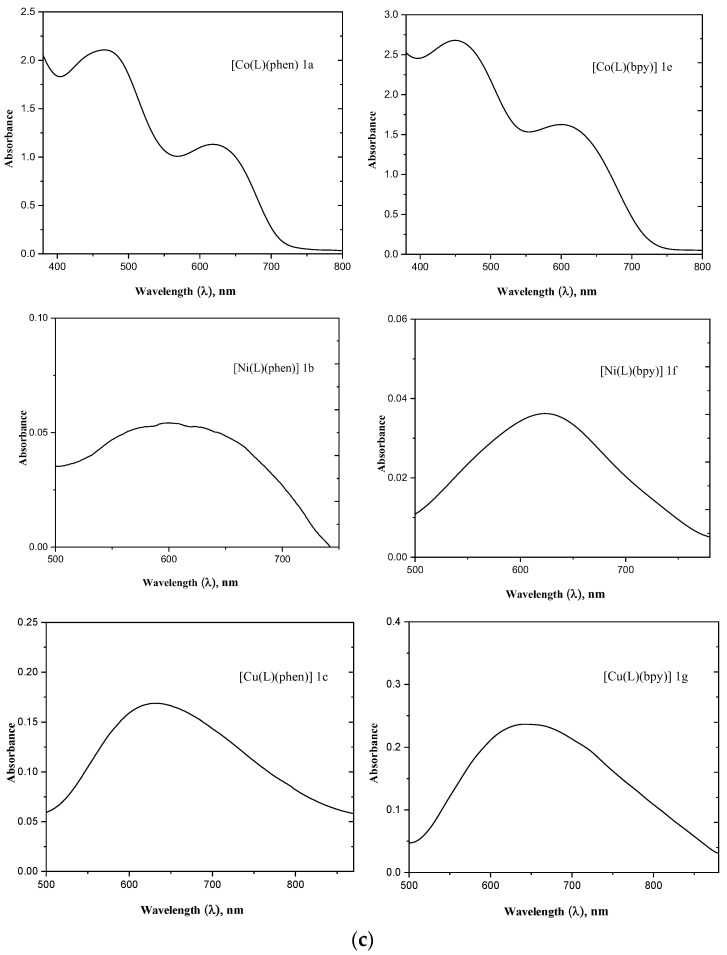
UV spectra of (**a**) complexes **1a**–**1d** and (**b**) complexes **1e**–**1h** and (**c**) visible spectra of complexes **1a**–**1h** in methanol.

The electronic spectra of metal(II) complex **1**(**a**–**h**) were measured using methanol solution (Figure 1a–c) and are presented in Table 2. A series of intense bands were observed for the ligand (HL) at 264, 353, and 409 nm, which were assigned to π–π* and n–π* transitions involved in azomethine nitrogen and aromatic ring systems, and this transition showed a decrease in wavelength at 230–300 nm upon complexation [29]. A moderately intense band at around 330–390 nm corresponds to the ligand-to-metal charge transfer transition [30]. In the visible region, cobalt(II) complexes **1a** and **1e** showed two weak absorption bands at 494 and 634 nm as well as 463 and 619 nm, respectively, suggesting a distorted trigonal bipyramidal geometry around the metal ion [31]. Whereas the nickel(II) complexes **1b** and **1f** and copper(II) complexes **1c** and **1g** showed one weak broad absorption peak in the 620–650 nm region due to the distorted square pyramidal geometry around the metal(II)complexes [32]. Moreover, the zinc(II) complexes **1d** and **1h** did not present any bands in the visible region due to their diamagnetic behavior and the d^10^ electronic configuration of the metal ion [33].

#### 3.2.3. Mass Spectral Analysis

The ESI^+^ mass spectra of complexes **1a** and **1b** (Figure S2) were determined to have molecular peaks at *m/z* = 536.50 and 536.20, which were attributed to the [C_24_H_20_BrCoN_3_O_3_)]^+^ and [C_24_H_20_BrNiN_3_O_3_)]^+^ fragment ions, respectively. These complexes show a base peak at *m/z* = 355.19 and 354.11 due to the fragmentation of [C_12_H_12_BrCoNO_3_]^2+^ and [C_12_H_12_BrNiNO_3_]^2+^ ions (Figures S1 and S2). In the same way, other metal(II) complexes (**1c**–**1h**) exhibited molecular ion peaks at *m/z* = 541.78, 543.28, 513.03, 512.89, 517.69, and 519.38, which corresponded to [M^1c−1h^ (L)(diimine)]^+^ ions (M=Co, Ni, Cu, and Zn). The resulting spectral data of the complexes showed the formation of the suggested molecular structure.

#### 3.2.4. Thermal Analysis

**Figure 2:**
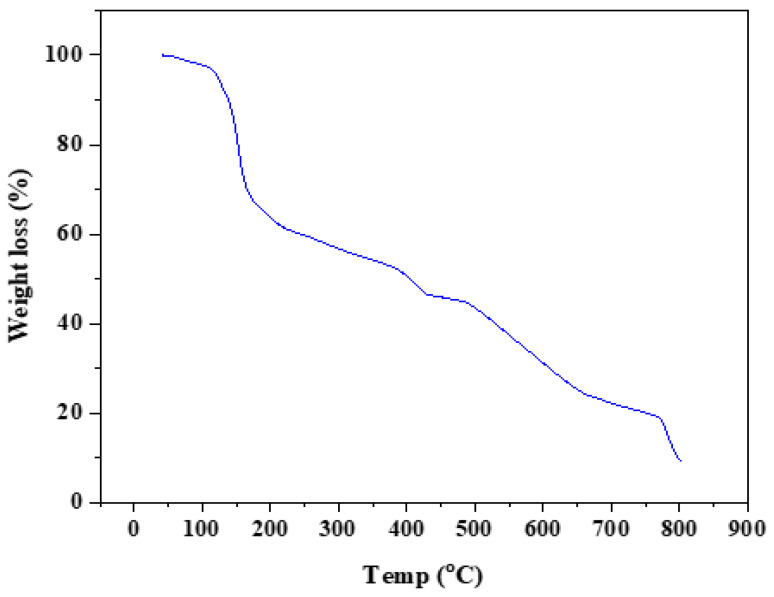
TGA curve of [Cu(L)(bpy)] **1g** complex.

The thermal behaviors of the metal complexes were determined under a nitrogen atmosphere, as shown in Figure 2. Upon comparison, it is clear that the weight loss of the metal(II) complexes correlates with different compositions at specific temperatures. The [Cu(L)(bpy)] **1g** complex displays a weight decrement in three steps from 38 to 800 °C. The first one is related to the decrement in the outer lattice dehydration of a single H_2_O molecule at 30–120 °C. The second step temperature range of 120–270 °C is due to the loss of organic molecules of C_10_H_8_N_2_ (2,2′-bipyridyl). A peak corresponding to a weight loss of 24.52% (calcd. 24.46%) at 420–510 °C was related to the ligand moiety in the metal complexes in the third step and the subsequent complex, thus leaving CuO as a residue. The same trend was traced in the TGA plots of the other metal complexes.

#### 3.2.5. EPR Spectral Analysis

**Figure 3:**
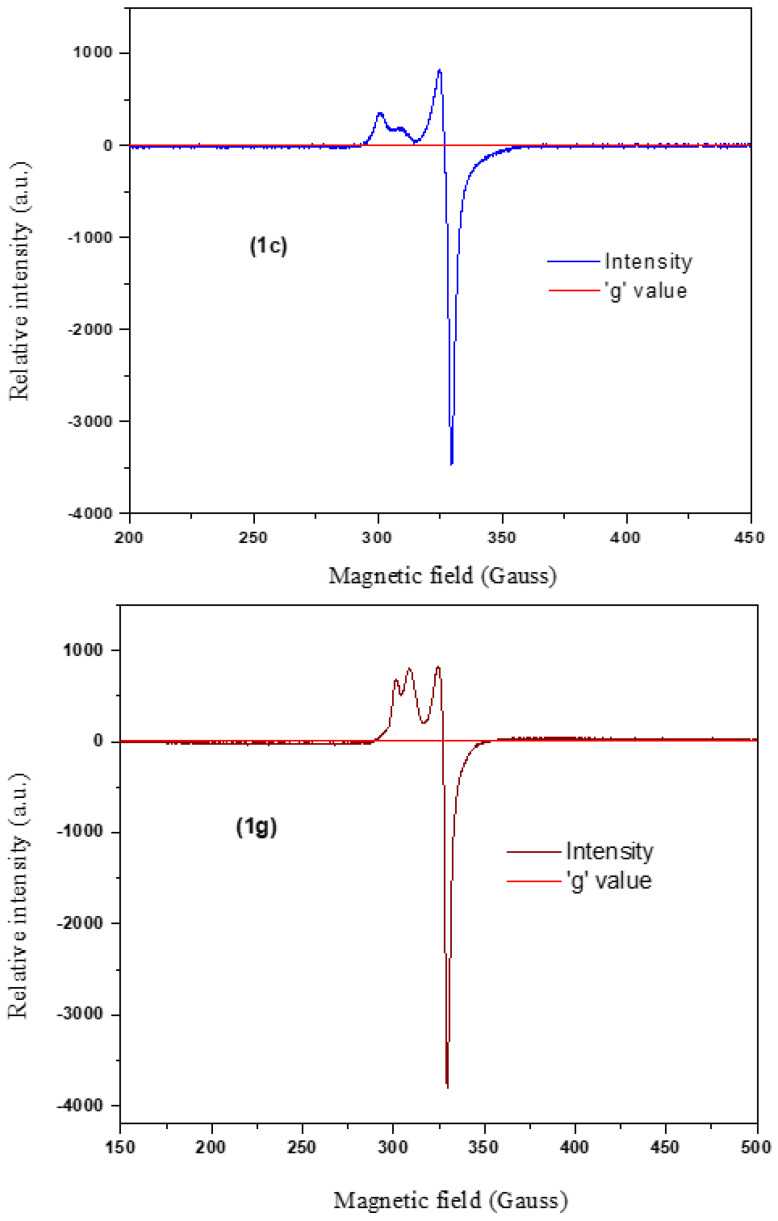
X−band EPR spectrum of complex [Cu(L)(phen)] **1c** and [Cu(L)(bpy)] **1g** at room temperature.

The EPR spectra of the copper(II) complexes [Cu(L)(phen)] **1c** and [Cu(L)(bpy)] **1g** were measured in polycrystalline conditions at 298 K. The hyperfine splitting patterns of the EPR spectra revealed that the discrete *g_||_* and *g_⊥_* values obtained correspond to axial spectral features (Figure 3). The spectral data suggested that unpaired electrons of the copper(II) complex lay in the d_x_^2^_–y_^2^ molecular orbital having a ground state((^2^B_1g_) with *g_||_* > *g_⊥_* > 2.0023. The g_||_ magnitudes elucidated the types of bonds (ionic or covalent) included in the observed metal-to-ligand coordination process. Usually, ionic bonds showed *g_||_*> 2.4 and covalent bonds indicated *g_||_* < 2.4. The *g_||_*values of complexes **1c** and **1g** were 2.184 and 2.186, thus confirming the covalent nature of the copper(II) complexes. The attained g values confirmed the square pyramidal geometry of the copper complexes reported in previous studies [34]. According to Hathway [35], the geometric parameter ‘G’ indicates an exchange interaction between the copper–copper centers and is calculated as follows:

G = (*g_||_* − 2)/(*g_⊥_*− 2) for axial spectra

The G values calculated for complexes **1c** and **1g** were 2.61 and 3.064, respectively. As these G values were found to be around 3.0, the presence of a d_x_^2^_–y_^2^ ground state of a square pyramidal geometry and a substantial exchange interaction in the polycrystalline state were confirmed [36,37].

#### 3.2.6. XRD Analysis

**Figure 4:**
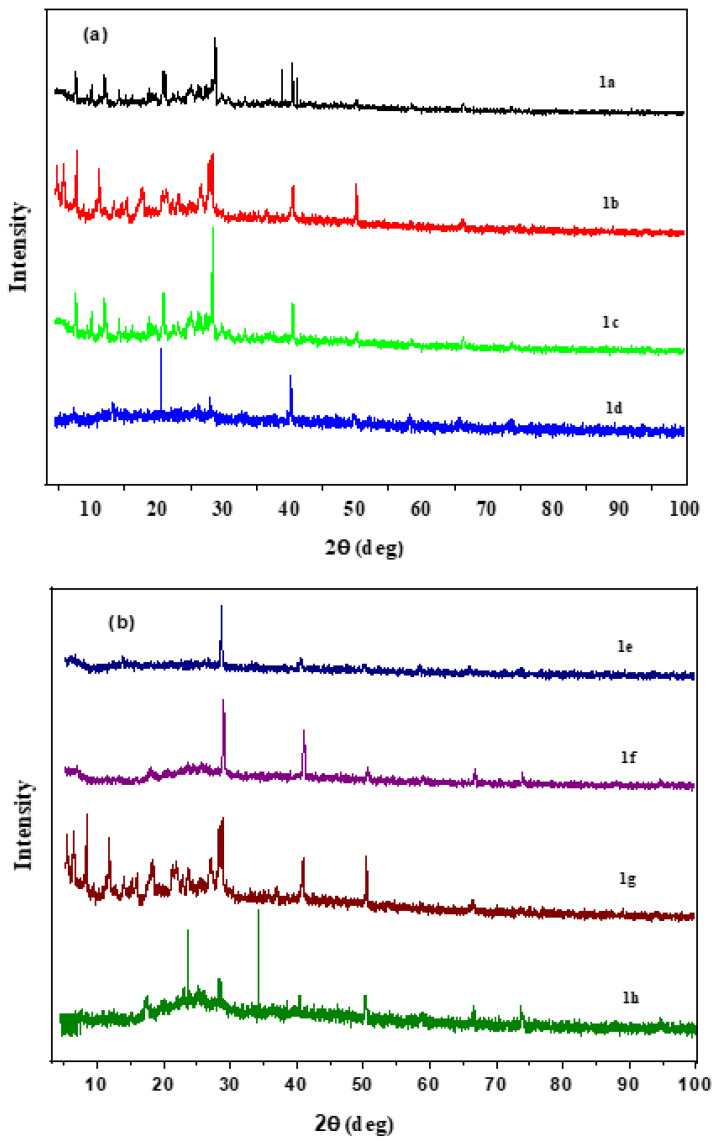
Powdered X-ray patterns of (**a**) complexes **1a**–**1d** and (**b**) complexes **1e**–**1h**.

The purity, crystallite size, and crystallinity of the synthesized complexes were analyzed by Powder XRD [38,39,40,41]. The diffractograms of complexes **1a**, **1b**, **1f**, and **1h** are shown in Figure 4a,b. In the region 5–50° (2θ), complexes **1**(**a**–**c**), and **1g** showed sharp diffraction patterns, suggesting that the complexes exist in a crystalline structure. While complexes **1**(**d**–**f**), and **1h** showed very few diffraction peaks, they indicated that the complexes were between crystalline and amorphous phases [42]. The crystallite sizes of the samples were evaluated using Scherrer’s equation [43,44]. From the calculated data, the average crystallite size of complexes is in the range of 50–60 nm.

### 3.3. Electrochemical Studies

The redox potential for metal(II) complexes **1**(**a**–**h**) was calculated via cyclic voltammetry in DMF containing 0.1 M of tetra(n-butyl) ammonium perchlorate and at a scan rate of 100 mVs^−1^, for which the results are listed in Table 3. The absence of a cyclic voltammogram for complexes **1d** and **1h** was due to the electrochemically inactive nature of the zinc(II) ion. The anodic and cathodic peak current ratios (Ip_c_/Ip_a_) were found to be less than unity, thereby suggesting that the metal(II) complexes were irreversible during the redox process.

#### 3.3.1. Reduction Process

**Figure 5:**
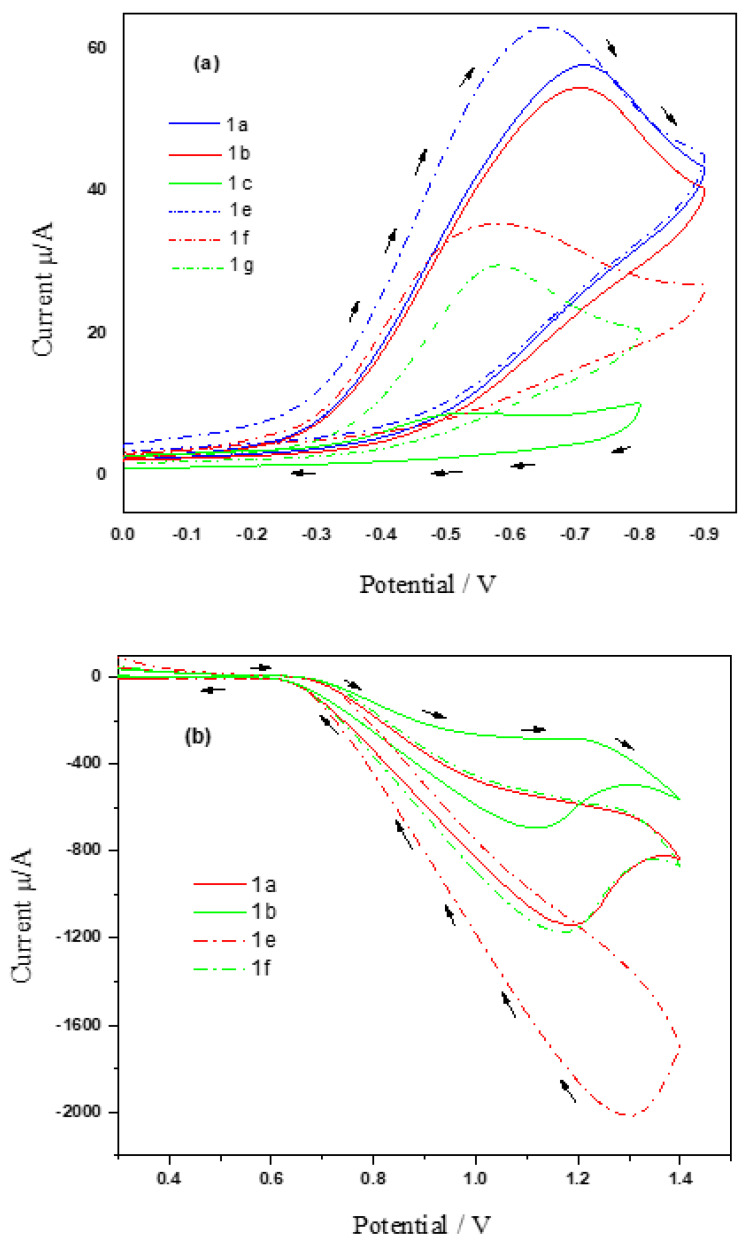
Cyclic voltammograms of metal(II) complexes **1a**–**h**. (**a**) Reduction process at cathodic region. (**b**) Oxidation process at anodic region in DMF containing 0.1M of tetra(n−butyl)ammoniumperchlorate. The scan rate applied was 100 mVs^−1^.

The cyclic voltammograms of the Co(II), Ni(II), and Cu(II) metals were studied at the cathodic region from 0 to –1.4 V (Figure 5a). The voltammogram peaks of cobalt(II) complex **1a**, nickel(II) complex **1b**, and copper(II) complex **1c** showed irreversible one-electron transfer reduction at −0.71 V, −0.71 V, and −0.54 V. Likewise, the absence of counter-reduction patterns in the reverse scan corroborated the irreversible character of the metal in complexes **1**(**e**–**g**) [45,46]. These reduction potential peaks were observed at −0.66 V, −0.58 V, and −0.58 V, respectively. It was suggested that the following one-electron reduction method should be used:

$$\left[M^{\mathrm{II}}(L)(\mathrm{phen}\;\mathrm{or}\;\mathrm{bpy})]\overset{e^{-}}{\to }[M^{I}(L)(\mathrm{phen}\;\mathrm{or}\;\mathrm{bpy})\right]$$

#### 3.3.2. Oxidation Process

The cyclic voltammograms of the Co(II), Ni(II), and Cu(II) metals were studied at the cathodic region from 0 to –1.4 V (Figure 5b). The cyclic voltammograms for cobalt(II) complex **1a** and nickel(II) complex **1b** revealed irreversible transfer oxidation at +1.19 V and +1.12 V, respectively. Likewise, the cobalt(II) complex **1e** and nickel(II) complex **1f** showed oxidation potential at +1.30 and +1.17, respectively; finally, the absence of a counter-oxidation peak in the reverse pattern verified the irreversible nature of the metallic ions [47]. The oxidation step at the anodic region is defined as follows:

(1) $$\left[M^{\mathrm{II}}(L)(\mathrm{phen}\;\mathrm{or}\;\mathrm{bpy})]\overset{{-e}^{-}}{\to }[M^{\mathrm{III}}(L)(\mathrm{phen}\;\mathrm{or}\;\mathrm{bpy})\right]$$

### 3.4. Catalytic Activity

**Scheme 2:**
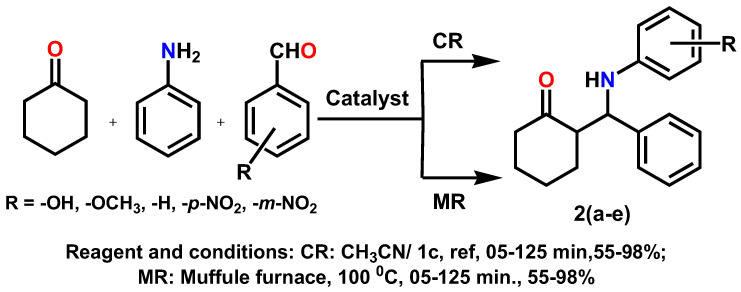
Catalytic one-pot three-component Mannich reaction through multiple approaches.

Subsequently, all metal(II) complexes utilized for multi-component reactions in a single pot were examined at various concentrations with or without a solvent (Scheme 2). To improve the catalytic performance of Schiff base metal(II) complex **1c**, substituted β-amino carbonyl derivatives were synthesized in one pot repeatedly at different concentrations, including 0.0384 mmol, 0.0769 mmol, 0.1154 mmol, and 0.1539 mmol. While floating the amount of the catalyst from 0.1154 mmol to 0.1539 mmol, the yield rate and reaction time remained unaffected.

Various derivatives of substituted β-amino carbonyl compounds were synthesized using conventional and solid-phase techniques, for which reactions were continued at multiple varying concentrations. These results are shown in Table 4 and Table 5. In the conventional technique, the zinc enolate is produced by linkage, and the catalyst then deprotonates the alkynyl ketone. However, the reaction time and percentage of yield are notable for the pricey Zn-Pro Phenol complex catalyst. *β*-Amino carbonyl groups were synthesized with less than 0.5 mmol of a nanocomposite catalyst, for which more than 10 h were required to complete the investigation. Furthermore, 60 mg of metal (II) salt was used for 5 min under the silica-supported method.

The synthesized metal (II) complexes were utilized as a model catalyst for the acceleration of the Mannich reaction. Under the same reaction conditions, the catalyst was used up to four times in the synthesis of substituted β-amino carbonyl compounds. The final product was the same as when it was first used, even after the fourth recycling phase, as shown in Table 6.

### 3.5. Theoretical Studies

#### 3.5.1. Geometry Optimization

**Figure 6:**
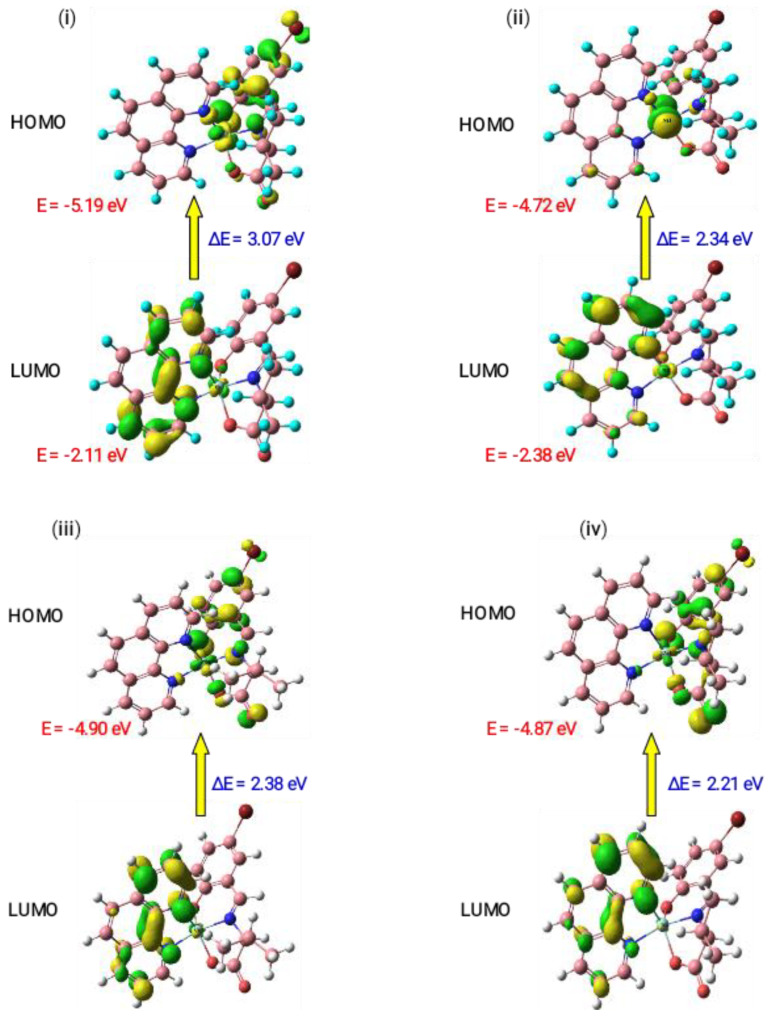
Frontier molecular orbitals of investigated complexes, namely, (**i**) cobalt(II) complex **1a**, (**ii**) nickel(II) complex **1b**, (**iii**) copper(II) complex **1c**, and (**iv**) zinc(II) complex **1d**, using B3LYP/LACVP++ basis set.

**Figure 7:**
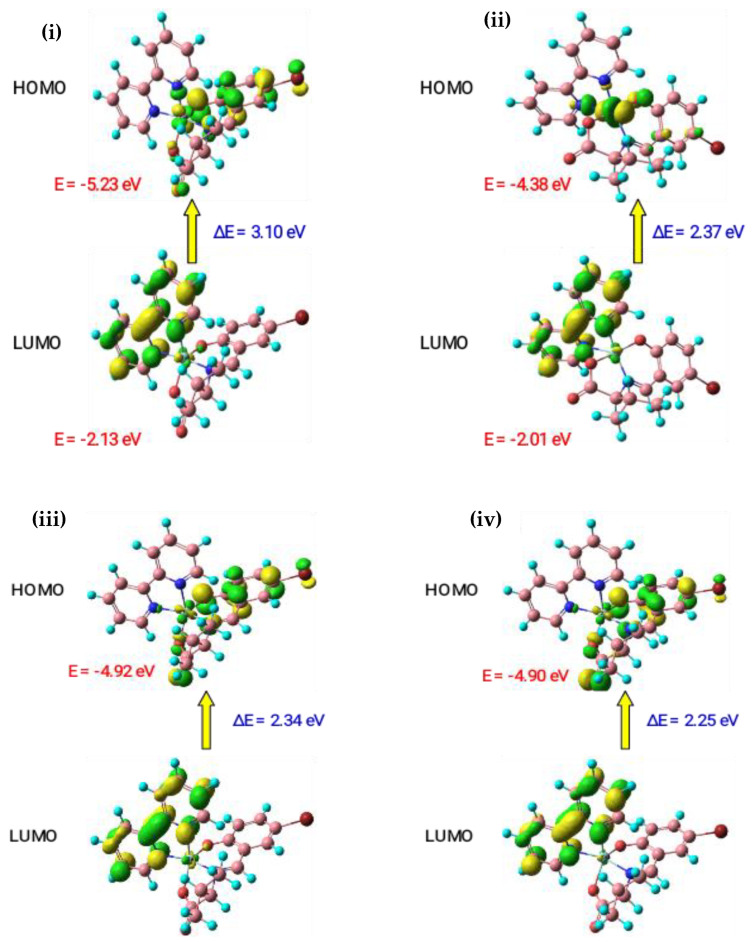
Frontier molecular orbitals of investigated complexes, namely, (**i**) cobalt(II) complex **1e**, (**ii**) nickel(II) complex **1f**, (**ii**) copper(II) complex **1g**, and (**iv**) zinc(II) complex **1h**, using B3LYP/LACVP^++^ basis set.

With the help of the Jaguar 8.8 software, which is based on DFT with the B3LYP and LACVP++ basis sets, the optimal geometry for the metal(II) complexes was obtained. The chosen bond angles and bond lengths are shown in Table 7 and Table 8, and the molecular structures of the metal(II) complexes are illustrated in Figure 6 and Figure 7. It was discovered that the computed values of bond angles and bond lengths were more suitable for forecasting the geometry of the metal(II) complexes. The geometric parameter ‘*τ*’ determined the molecular structure of the complex for the pentacoordinate system, and it was computed using the following formula [48].

*τ* = (*β* − *α*)/60 (2)

In the formula presented above, *α* and *β* are axial and equatorial bond angles, respectively. When *τ* ≈ 0, the geometry becomes square pyramidal, and it becomes closer to 1 for a perfect trigonal bipyramidal structure. For a trigonal bipyramidal geometry, the *τ* values lie between 0 and 0.5. In this study, the calculated bond angles for complexes **1**(**a**–**h**), were 171.2, 156.9, 154.9, 151.1, 172.8, 162.2, 152.7, and 151.9°, respectively, which correspond to β (axial bond angles), and the values 126.2, 153.4, 149.1, 135.7, 125.1, 158.5, 148.6, and 134.8°, respectively, which correspond to α (equatorial bond angles). The calculated *τ* values for the metal(II) complexes were 0.75, 0.05, 0.09, 0.26, 0.79, 0.06, 0.06, and 0.28, respectively. Complexes **1a** and **1e** and were, as suggested, of a trigonal bipyramidal geometry due to their τ values of 0.75 and 0.79, respectively. Complexes **1b**, **1c**, **1f**, and **1g** were suggested to possess a square pyramidal geometry because their τ values were very close to 0 (0.05–0.09). Finally, complexes **1d** and **1h** were suggested to possess a distorted trigonal bipyramidal geometry due to their *τ* values of 0.26 and 0.28, respectively.

#### 3.5.2. Molecular Orbital Analysis

The molecular orbitals for the synthesized metal(II) complexes were theoretically analyzed by frontier molecular orbital theory. This theory states that the interaction between the frontier orbitals (HOMO and LUMO) of the reacting molecules generate an electric change. The energies of the primary orbitals were used to determine whether a compound would be stable or chemically reactive at the lowest unoccupied molecular orbital (LUMO) and the highest occupied molecular orbital (HOMO). A tendency to donate an electron corresponds to the HOMO, while a tendency to withdraw an electron corresponds to the LUMO [49]. In many molecular systems, the energy difference (*ΔE*) between the HOMO and LUMO is thought to be a crucial stability index for highlighting structural and conformational barriers. Charge transfer between the HOMO and LUMO happens when *ΔE* is low. The increased activity of the molecule is influenced by the lower *ΔE* value.

In this study, the significant quantum parameters, such as the HOMO–LUMO energy gap (*ΔE*), chemical potentials (*Pi*), absolute electronegativities (*χ*), absolute softness ((*σ*), absolute hardness (*η*), global electrophilicity (*ω*), global softness (*S*), and additional electronic charge (*Δ_Nmax_*), were calculated in accordance with Koopman’s hypothesis using the following equations [50]:

*χ* = −(*E_HOMO_* + *E_LUMO_*)/2

*η* = *E_LUMO_* − *E_HOMO_*/2

*ΔE* = *E_LUMO_* − *E_HOMO_*

*σ* = 1/*η*

*S* = 1/2*η*

*Pi* = −*χ*

Ω = *Pi*^2^/2*η*

*ΔN_max_* = −*Pi/η*

The obtained parameters for the complexes are given in Table 9. The Δ*E* values of complex **1**(**a**–**h**) were found to be 3.07, 2.34, 2.38, 2.21, 3.10, 2.37, 2.34, and 2.25 eV, respectively. Particularly, the zinc(II) complexes **1d** and **1h** have the lowest energy band gap, suggesting higher activity and lesser stability. The lowest energy gap value of the zinc(II) complex **1d** demonstrated an undemanding electronic transition between the HOMO and LUMO, which may be reason for the higher bioactivity observed.

### 3.6. Molecular Docking Studies

#### Validation of the Active Site of Thymidylate Synthase

**Figure 8:**
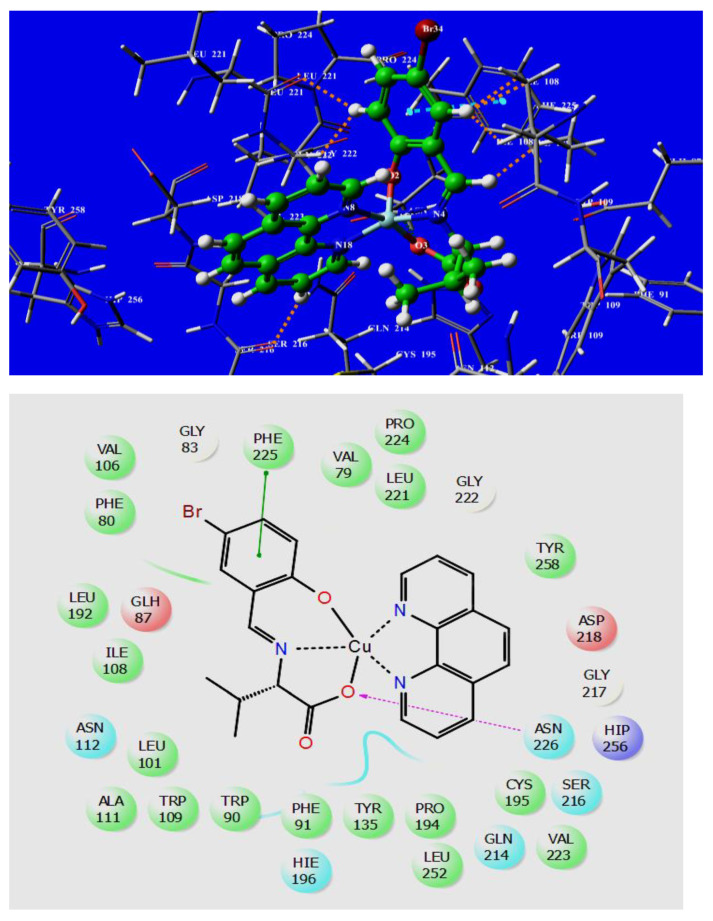
The 3D and 2D interactions of [Cu(L)(phen)] complex **1c** located in hydrophobic sites of thymidylate synthase receptor.

Thymidylate synthase (TS) plays a decisive role in the biosynthesis of DNA precursors. An increased level of thymidylate synthase activity has been observed in colorectal, breast, cervical, kidney, and lung tumors. Many researchers have encountered difficulties when attempting to find a better way to regulate thymidylate synthase action for both the development of therapeutic strategies and tumor prevention. Hence, the prepared complexes were assessed with respect to the binding affinity of the thymidylate synthase receptor to understand the interactive behavior of the studied complexes with the TS receptor and thus develop a strategy for their optimization. By analyzing the metal(II) complexes **1**(**a**–**h**) applied for docking with thymidylate synthase (PDB ID: 1HZW) and their binding affinity values, it was revealed that the complexes were more active on that site. The calculated docking scores and active sites of TS with various modes of interaction are shown in Table 10 (Figures S3 and S4 from Supplementary Materials). The docking study’s results revealed that all the complexes were located within the hydrophobic site of the TS receptor. The docking scores for the metal(II) complexes with a TS receptor were found to be −5.70, −5.615, −5.791, −5.367, −5.49, −4.97, −5.026, and −5.223 kcal mol^−1^. In addition, it was found that complex 1c had the highest docking score due to binding interaction with the TS receptor via π–π stacking, hydrogen bonding, and hydrophobic interactions, as shown in Figure 8. This complex showed one hydrogen bond (distance of 2.33 Å) interaction between an amino hydrogen of residue ASN 226 with the carboxylate oxygen of the complex. Further, complex **1c** showed π–π stacking interaction between the residue PHE 225 and a phenolate ring with an interaction distance of 4.01 Å. Furthermore, the complex showed an abundance of hydrophobic interactions with different amino acid residues, such as VAL 79, PHE 80, TRP 90, PHE 91, LEU 101, VAL 106, ILE 108, TRP 109, ALA 111, TYR 135, LEU 192, PRO 194, CYS 195, PRO 194, LEU 221, VAL 223, PRO 224, PHE 225, and TYR 258. The prepared metal(II) complexes showed better activity compared to that presented in our previously published article [51]. The highest docking score value of complex **1c** was obtained with thymidylate synthase, which encouraged us to study the cytotoxicity of the metal complexes experimentally.

### 3.7. Biological Evaluation

#### 3.7.1. In Vitro Antibacterial Assay

**Figure 9:**
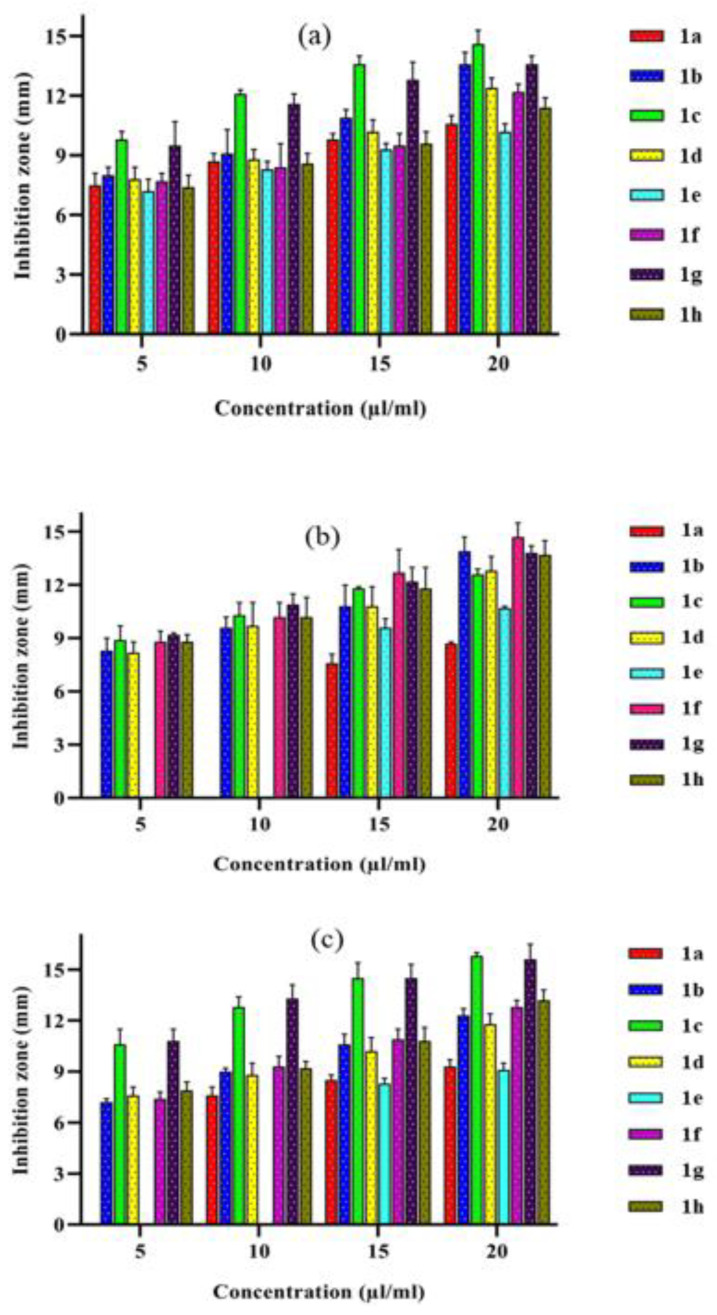
Antibacterial activity of metal(II) complexes (**1a**–**1h**) tested against (**a**) *Escherichia coli*, (**b**) *Klebsiella pneumonia*, and (**c**) *Staphylococcus aureus* bacteria at different concentrations.

In vitro antibacterial activity for metal(II) complex **1**(**a**–**h**) was checked against two Gram-negative bacteria (*Escherichia coli* and *Klebsiella pneumonia*) and one Gram-positive bacterium (*Staphylococcus aureus*) in different concentration ranges from 5–20 µL/mL using the agar diffusion method [21,52,53]. The gradient changes were illustrated as graphs (Figure 9a–c), and their data are shown in Table 11. From the data, it is evident that the metal complexes showed better activity upon an increase in concentration.

The results showed that all the metal complexes have considerable activity at a concentration of 20 µL/mL. Among them, complexes **1c** and **1f** had higher activity at this concentration against the microbial test strains, specifically with respect to copper(II) complex **1c** against *Escherichia coli*, a group of Gram-negative bacteria, and *Staphylococcus aureus*, a Gram-positive bacterium, for which there were greater inhibition zones at 14.6 and 15.8 mL, respectively. Further, complex **1f** was more active against *Klebsiella pneumonia* (a Gram-negative bacterium), having a higher inhibition zone at 14.7 ± 0.8 µL/mL. From the above particulars, it is evident that the metal ions coordinated with diamine play a key role in antimicrobial activity, and we conclude that metalation is significantly related to the inhibitory activity of complexes as explained by Tweedy’s chelation theory [54]. This increasing activity showed that chelation significantly improved the lipophilicity of the chemicals, resulting in their absorption through the lipoid layer of the microbe’s cell membrane and DNA destruction [55,56]. These results are in agreement with those presented in previous studies involving L-alanine-incorporated metal(II) complexes [56,57].

#### 3.7.2. In Vitro Anticancer Activity

**Figure 10:**
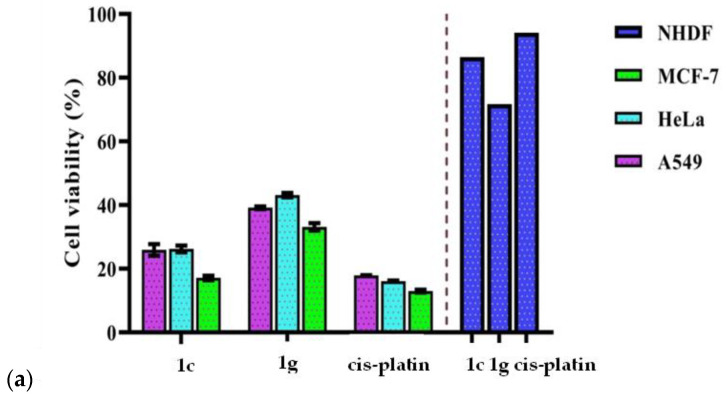

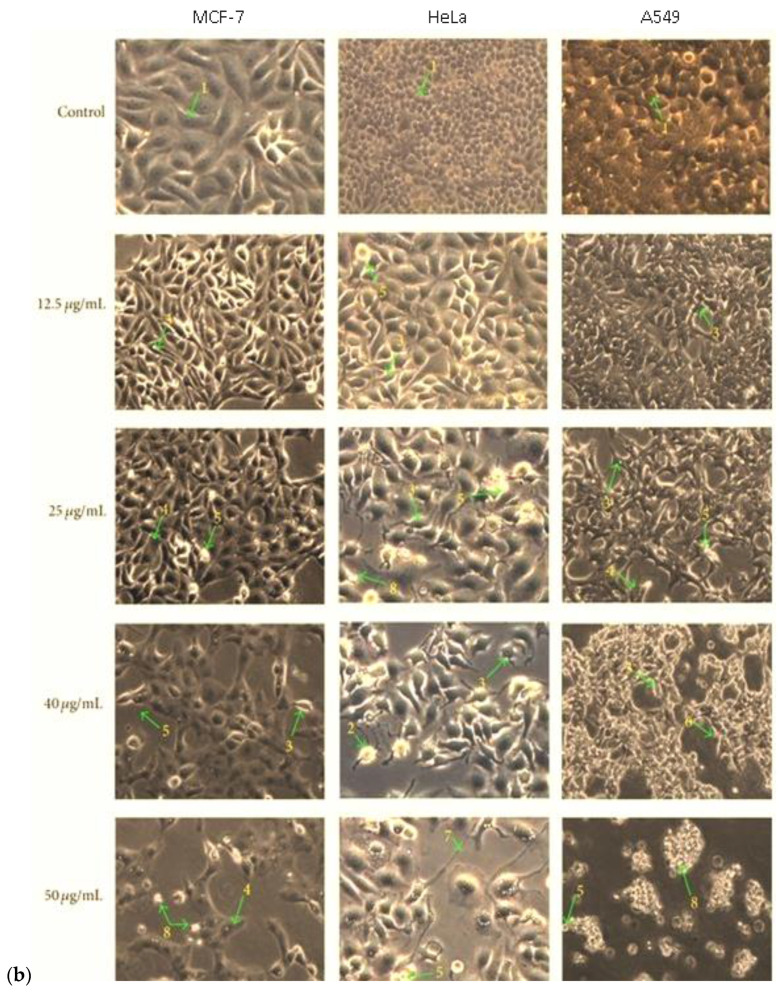
(**a**) In vitro anticancer activity of copper(II) complexes **1c** and **1g** against A549, HeLa, and MCF-7 cancer cell lines. (**b**) MTT-based antiproliferative photomicrographs of copper(II) complex **1c** on MCF7 HeLa and A549 cancer cell lines after 24 h incubation under a phase contrast microscope. Arrows indicate (1) control cell, (2) condensed nuclei, (3) cell shrinkage, (4) membrane blebbing, (5) apoptotic bodies, (6) bubbling, and (7) echinoid spikes.

The metal Cu(II) complexes **1c** and **1g** were used in the MTT procedure to measure the vitality of the analyzed cells. Different cancer cell lines were used for the MTT assay, such as an A549 lung cancer cell line, a HeLa cervical cancer cell line, an MCF-7 breast cancer cell line, and NHDF cell lines, while employing *cis*-platin as a control. Their IC_50_ values for each complex were determined up to 24 h alongside an increase in complex concentration. The data have been presented in a graph (Figure 10a) and in Table 12. The IC_50_ value of complex **1c** showed 25.95 ± 1.82, 26.26 ± 1.06, and 17.13 ± 0.74 μg/mL for the A549, HeLa, and MCF-7 cell lines, respectively. From the results, complex **1c** showed a higher anticancer efficacy than complex **1g**, and its value was found to be very close to that of *cis*-platin. The photomicrographs of complex **1c** against MCF-7 showed different cytotoxic morphologies of condensed nuclei, cell shrinkage, membrane blebbing, apoptotic bodies, bubbling, and echinoid spikes (Figure 10b). The toxicity results also suggested that complex **1c** is less toxic than complex **1g** with respect to the NHDF cell line, for which the following order was observed: MCF-7 > A549 > HeLa. The extended aromatic conjugation of the phenanthroline moiety and strong hydrophobic interaction of complex **1c** showed higher potential anticancer activities compared to the bipyridyl co-ligand.

## 4. Conclusions

In this work, Schiff base ligands (HL) and heterocyclic bases (1,10-phenanthroline or 2,2′-bipyridyl) were used to synthesize mixed-ligand metal(II) complexes **1**(**a**–**h**). Based on the spectral results, it was determined that the Co(II), Ni(II), Cu(II), and Zn(II) complexes assumed the geometry of a distorted square pyramidal shape. With regard to the electrochemical behavior of the complexes, it was observed that one electron was irreversibly transferred. The results regarding catalytic activity showed that the muffle furnace approach reached the target molecule much faster than the conventional method. Metal(II) complex **1c** revealed significant antibacterial activity against *Escherichia coli* and *Staphylococcus aureus* at a 20 µg/mL concentration applied using the agar diffusion method. Similarly, the in vitro cytotoxicity of complex [Cu(L)(phen)] **1c** showed promising anticancer activity against MCF-7 (17.13 ± 0.74), A549 (25.95 ± 1.82), and HeLa (26.26 ± 1.06) cancer cell lines, whose IC_50_ values were very close in terms of activity to that of cis-platin. The frontier orbital (HOMO–LUMO) analyses revealed that the complex [Zn(L)(phen)] **1d** exhibited a lower band gap (2.21 eV) than the other complexes. The smaller energy gap led to an easing of the transition occurring between energy levels and precipitated the higher biological activity of the metal(II) complexes. Finally, the docking score of complex **1c** (−5.791 kcal mol^−1^) showed better binding interactions with the thymidylate synthase receptor, viz., several hydrophobic, stacking, and hydrogen bonding interactions. Further investigation and developments in the area of Schiff base complexes of transition metal ions would be highly beneficial for industries and drug-related research.

## Figures and Tables

**Table 1:** Physical and analytical data of metal(II) complexes **1**(**a**–**h**).

Complexes	Formula	Color	Mol.Wt. gm/mol	M.p. (°C)	Yield (%)	Found (calc.) (%)		
						C	H	N
[Co(L)(phen)] **1a**	C_24_H_20_BrCoN_3_O_3_	Brown	537.08	>300	80	53.63 (53.65)	3.72 (3.75)	7.81 (7.82)
[Ni(L)(phen)] **1b**	C_24_H_20_BrN_3_NiO_3_	Pale green	536.92	>300	80	53.66 (53.68)	3.73 (3.75)	7.80 (7.82)
[Cu(L)(phen)] **1c**	C_24_H_20_BrCuN_3_O_3_	green	541.78	>300	88	53.19 (53.20)	3.70 (3.72)	7.72 (7.75)
[Zn(L)(phen)] **1d**	C_24_H_20_BrN_3_O_3_Zn	Pale yellow	543.28	>300	67	52.98 (53.02)	3.69 (3.71)	7.72 (7.73)
[Co(L)(bpy)] **1e**	C_22_H_20_BrCoN_3_O_3_	Brown	513.03	>300	84	51.46 (51.48)	3.92 (3.93)	8.18 (8.19)
[Ni(L)(bpy)] **1f**	C_22_H_20_BrN_3_NiO_3_	Pale green	512.89	>300	79	51.49 (51.51)	3.91 (3.93)	8.17 (8.19)
[Cu(L)(bpy)] **1g**	C_22_H_20_BrCuN_3_O_3_	green	517.69	>300	83	51.00 (51.02)	3.88 (3.89)	8.09 (8.11)
[Zn(L)(bpy) ] **1h**	C_22_H_20_BrN_3_O_3_Zn	Pale yellow	519.38	>300	67	50.81 (50.84)	3.86 (3.88)	8.06 (8.09)

**Table 2:** FT-IR and UV–visible spectral data of mixed ligand metal(II) complexes **1**(**a**–**h**).

Complexes	FT-IR Spectral Data (cm^−1^)					UV–Visible Spectral Data (λ_max_/nm (ε/M^−1^cm^−1^dm^3^)	
	ν(-C=N-)	ν_as_(COO)	ν_s_(COO)	ν(M-O)	ν(M-N)	d-d	Charge Transfer
HL	1643	1594	1405	--	--	--	409 (500), 353 (180), 264 (1590)
[Co(L)(phen)] **1a**	1636	1587	1371	549	457	494 (152) 634 (58)	228 (33,895) (π–π*) 269 (25,834) (n–π*) 337 (1209) (LMCT)
[Ni(L)(phen)] **1b**	1612	1586	1380	542	460	623 (6)	254 (57,302) (π–π*) 288 (36,331) (n–π*) 389 (3657) (LMCT)
[Cu(L)(phen)] **1c**	1611	1562	1356	554	486	637 (17)	259 (864,222) (π–π*) 284 (58,665) (n–π*) 372 (1502) (LMCT)
[Zn(L)(phen)] **1d**	1622	1557	1376	549	502	--	270 (35,064) (π–π*) 292 (9154) (n–π*) 373 (4019) (LMCT
[Co(L)(bpy)] **1e**	1623	1575	1369	562	488	463 (127) 619 (65)	249 (19,579)(π–π*) 301 (8048) (n–π*) 389 (854) (LMCT)
[Ni(L)(bpy)] **1f**	1634	1570	1379	542	485	625 (4)	238 (4056) (π–π*) 298 (2094) (n–π*) 379 (920) (LMCT)
[Cu(L)(bpy)] **1g**	1594	1568	1358	553	461	642 (24)	240 (13,846) (π–π*) 300 (11,085) (n–π*) 375 (144) (LMCT
[Zn(L^3^)(bpy)] **3h**	1635	1577	1370	548	497	--	274 (20,198) (π–π*) 294 (16,748) (n–π*) 374 (8425) (LMCT)

**Table 3:** Electrochemical data of metal(II) complexes in DMF.

Complexes	Ep_c_ (V)	Ep_a_ (V)	∆Ep (V)	Ip_c_ (μA)	Ip_a_ (μA)	Ip_c_/Ip_a_
[Co(L)(phen)] **1a**	−0.71	+1.19	1.90	57.63	−1138	0.050
[Ni(L)(phen)] **1b**	−0.71	+1.12	1.83	54.50	−694	0.078
[Cu(L)(phen)] **1c**	−0.54	--	--	8.90	--	--
[Zn(L)(phen)] **1d**	NA *	NA	--	--	--	--
[Co(L)(bpy)] **1e**	−0.66	+1.30	1.96	62.97	−2015	0.031
[Ni(L)(bpy)] **1f**	−0.58	+1.17	1.75	35.40	−1174	0.030
[Cu(L)(bpy)] **1g**	−0.58	--	--	29.55	--	--
[Zn(L)(bpy)] **1h**	NA	NA	--	--	--	--

*: Not applicable.

**Table 4:** Synthesis of β-Amino carbonyl derivatives assisted by various concentrations of metal complex **1**(**a**–**d**).

S.No	Catalyst		Deri- vative	Concentration of Substituted Phenanthroline Metal Salts											
				0.0367 mmol				0.0735 mmol				0.1103 mmol			
				CR		MR		CR		MR		CR		MR	
				T	Y	T	Y	T	Y	T	Y	T	Y	T	Y
1	**Substituted phenanthroline metal (II) salts**	**1a**	**2a**	85	65	40	75	75	76	30	82	65	88	20	97
			**2b**	95	64	45	71	85	74	35	79	75	86	25	94
			**2c**	75	66	35	75	70	77	30	82	60	92	20	90
			**2d**	65	73	30	80	60	83	25	84	50	91	15	93
			**2e**	55	76	25	80	45	88	20	87	40	90	10	95
2		**1b**	**2a**	95	62	50	71	85	74	40	80	75	86	30	94
			**2b**	105	60	55	73	95	71	45	74	85	83	35	91
			**2c**	85	61	45	72	80	75	40	80	70	90	30	88
			**2d**	75	71	40	78	70	78	35	77	60	88	25	89
			**2e**	65	72	35	76	55	82	30	85	50	86	20	91
3		**1c**	**2a**	105	60	60	69	95	70	50	78	85	82	40	92
			**2b**	115	57	65	70	105	67	55	71	95	80	45	88
			**2c**	95	59	55	68	90	72	50	77	80	82	40	90
			**2d**	85	67	50	79	80	76	45	78	70	82	35	89
			**2e**	75	72	45	76	65	82	40	85	60	86	30	91
4		**1d**	**2a**	115	59	70	64	105	69	60	72	95	80	50	90
			**2b**	125	57	75	70	115	67	65	71	105	80	55	88
			**2c**	105	59	55	65	90	70	50	75	80	80	40	88
			**2d**	95	65	50	77	80	73	45	75	70	81	35	88
			**2e**	75	70	45	72	65	79	40	83	60	82	30	90

**Table 5:** Synthesis of β-Amino carbonyl derivatives assisted by various concentrations of complex **1**(**e**–**h**).

S.No	Catalyst		Deri- vative	Concentration of Substituted Pyridine Metal Salts											
				0.0384 mmol				0.0769 mmol				0.1154 mmol			
				CR		MR		CR		MR		CR		MR	
				T	Y	T	Y	T	Y	T	Y	T	Y	T	Y
1	**Substituted 2,2-bipyidyl metal (II) salts**	**1e**	**2a**	75	68	35	78	70	79	20	85	55	91	15	96
			**2b**	85	67	40	74	80	77	25	82	65	89	20	92
			**2c**	65	69	30	79	65	80	20	85	50	95	15	95
			**2d**	55	76	25	83	55	86	15	87	40	94	10	97
			**4e**	45	79	20	84	40	91	10	90	30	93	05	98
2		**1f**	**2a**	85	65	45	74	75	77	30	83	65	89	25	92
			**2b**	95	63	50	77	85	73	35	77	75	86	30	90
			**2c**	75	64	40	75	70	78	30	83	60	93	25	91
			**2d**	65	74	35	81	60	81	25	81	50	91	20	89
			**2e**	55	75	30	79	45	85	20	88	40	89	15	91
3		**1g**	**2a**	95	63	55	71	85	73	40	81	75	85	35	90
			**2b**	105	60	60	73	95	70	45	74	85	83	40	89
			**2c**	85	62	50	71	80	75	40	80	70	85	35	89
			**2d**	75	70	45	82	70	79	25	81	60	86	30	90
			**2e**	65	75	40	79	55	85	30	88	50	89	25	92
4		**1h**	**2a**	105	63	65	68	95	72	50	75	85	85	45	91
			**2b**	115	61	70	73	105	71	55	74	95	83	50	90
			**2c**	95	62	50	65	80	73	40	78	70	84	35	89
			**2d**	85	69	45	77	70	76	35	81	60	86	30	92
			**2e**	65	73	40	72	55	82	30	87	60	85	25	90

**Table 6:** Reusability of catalysts **1a**–**1h**.

Catalyst	Percentage of Recyclability of Catalyst			
	First Cycle	Second Cycle	Third Cycle	Fourth Cycle
**1a**	91	91	90	89
**1b**	89	89	87	87
**1c**	87	87	87	85
**1d**	92	91	91	90
**1e**	90	89	89	88
**1f**	88	88	88	86
**1g**	90	89	89	88
**1h**	89	88	88	86

**Table 7:** Selected optimized geometric parameters of metal(II) complex **1**(**a**–**d**) determined by B3LYP method using LACVP++ basis set.

Bond Angle (deg) B3LYP/LACVP++							
[Co(L)(phen)] 1a		[Ni(L)(phen)] 1b		[Cu(L)(phen)] 1c		[Zn(L^1^)(phen)] 1d	
N(18)-Co(1)-N(8)	84.536	N(18)-Ni(1)-N(8)	85.326	N(18)-Cu(1)-N(8)	81.547	N(18)-Zn(1)-N(8)	79.945
N(18)-Co(1)-N(4)	171.246	N(18)-Ni(1)-N(4)	153.435	N(18)-Cu(1)-N(4)	154.944	N(18)-Zn(1)-N(4)	151.161
N(18)-Co(1)-O(3)	85.923	N(18)-Ni(1)-O(3)	86.310	N(18)-Cu(1)-O(3)	85.065	N(18)-Zn(1)-O(3)	81.925
N(18)-Co(1)-O(2)	98.447	N(18)-Ni(1)-O(2)	113.717	N(18)-Cu(1)-O(2)	108.729	N(18)-Zn(1)-O(2)	113.265
N(8)-Co(1)-N(4)	98.118	N(8)-Ni(1)-N(4)	94.750	N(8)-Cu(1)-N(4)	95.945	N(8)-Zn(1)-N(4)	98.121
N(8)-Co(1)-O(3)	126.249	N(8)-Ni(1)-O(3)	156.955	N(8)-Cu(1)-O(3)	149.119	N(8)-Zn(1)-O(3)	135.705
N(8)-Co(1)-O(2)	101.038	N(8)-Ni(1)-O(2)	94.179	N(8)-Cu(1)-O(2)	95.100	N(8)-Zn(1)-O(2)	103.250
N(4)-Co(1)-O(3)	84.163	N(4)-Ni(1)-O(3)	83.387	N(4)-Cu(1)-O(3)	82.697	N(4)-Zn(1)-O(3)	79.707
N(4)-Co(1)-O(2)	92.301	N(4)-Ni(1)-O(2)	92.797	N(4)-Cu(1)-O(2)	94.313	N(4)-Zn(1)-O(2)	95.274
O(3)-Co(1)-O(2)	123.606	O(3)-Ni(1)-O(2)	108.843	O(3)-Cu(1)-O(2)	112.778	O(3)-Zn(1)-O(2)	121.039
**Bond distance (Å)**							
Co(1)-N(18)	1.870	Ni(1)-N(18)	1.836	Cu(1)-N(18)	1.934	Zn(1)-N(18)	2.029
Co(1)-N(8)	1.918	Ni(1)-N(8)	1.884	Cu(1)-N(8)	2.059	Zn(1)-N(8)	2.049
Co(1)-N(4)	1.871	Ni(1)-N(4)	1.845	Cu(1)-N(4)	1.912	Zn(1)-N(4)	2.004
Co(1)-O(3)	1.837	Ni(1)-O(3)	1.822	Cu(1)-O(3)	1.882	Zn(1)-O(3)	1.937
Co(1)-O(2)	1.918	Ni(1)-O(2)	2.014	Cu(1)-O(2)	1.932	Zn(1)-O(2)	1.873

**Table 8:** Selected optimized geometry parameters of metal(II) complexes **1**(**e**–**h**) by B3LYP method using LACVP++ basis set.

Bond Angle (deg) B3LYP/LACVP++							
[Co(L)(bpy)] 1e		[Ni(L)(bpy)] 1f		[Cu(L)(bpy)] 1g		[Zn(L)(bpy)] 1h	
N(16)-Co(1)-N(8)	83.244	N(16)-Ni(1)-N(8)	78.400	N(16)-Cu(1)-N(8)	80.539	N(16)-Zn(1)-N(8)	78.477
N(16)-Co(1)-N(4)	172.888	N(16)-Ni(1)-N(4)	120.752	N(16)-Cu(1)-N(4)	152.716	N(16)-Zn(1)-N(4)	151.914
N(16)-Co(1)-O(3)	86.817	N(16)-Ni(1)-O(3)	79.803	N(16)-Cu(1)-O(3)	85.620	N(16)-Zn(1)-O(3)	82.922
N(16)-Co(1)-O(2)	97.946	N(16)-Ni(1)-O(2)	105.384	N(16)-Cu(1)-O(2)	113.072	N(16)-Zn(1)-O(2)	112.970
N(8)-Co(1)-N(4)	99.500	N(8)-Ni(1)-N(4)	158.557	N(8)-Cu(1)-N(4)	96.460	N(8)-Zn(1)-N(4)	98.792
N(8)-Co(1)-O(3)	125.104	N(8)-Ni(1)-O(3)	87.110	N(8)-Cu(1)-O(3)	148.646	N(8)-Zn(1)-O(3)	134.815
N(8)-Co(1)-O(2)	101.888	N(8)-Ni(1)-O(2)	88.367	N(8)-Cu(1)-O(2)	97.421	N(8)-Zn(1)-O(2)	104.122
N(4)-Co(1)-O(3)	83.670	N(4)-Ni(1)-O(3)	87.159	N(4)-Cu(1)-O(3)	81.407	N(4)-Zn(1)-O(3)	79.510
N(4)-Co(1)-O(2)	92.045	N(4)-Ni(1)-O(2)	94.828	N(4)-Cu(1)-O(2)	94.212	N(4)-Zn(1)-O(2)	94.892
O(3)-Co(1)-O(2)	122.850	O(3)-Ni(1)-O(2)	162.290	O(3)-Cu(1)-O(2)	117.930	O(3)-Zn(1)-O(2)	121.051
**Bond distance (Å)**							
Co(1)-N(16)	1.855	Ni(1)-N(16)	2.289	Cu(1)-N(16)	1.929	Zn(1)-N(16)	2.026
Co(1)-N(8)	1.897	Ni(1)-N(8)	1.865	Cu(1)-N(8)	2.002	Zn(1)-N(8)	2.030
Co(1)-N(4)	1.880	Ni(1)-N(4)	1.790	Cu(1)-N(4)	1.928	Zn(1)-N(4)	2.013
Co(1)-O(3)	1.840	Ni(1)-O(3)	1.805	Cu(1)-O(3)	1.890	Zn(1)-O(3)	1.933
Co(1)-O(2)	1.917	Ni(1)-O(2)	1.804	Cu(1)-O(2)	1.938	Zn(1)-O(2)	1.873

**Table 9:** The calculated quantum parameters for the metal(II) complex **1**(**a**–**h**).

Complexes	HOMO (eV)	LUMO (eV)	ΔE (eV)	*χ*	*η*	*σ*	*Pi*	*S*	*ω*	ΔN Max
[Co(L)(phen)] **1a**	−5.19	−2.11	3.07	3.65	1.54	0.65	−3.65	0.33	10.24	−2.38
[Ni(L)(phen)] **1b**	−4.72	−2.38	2.34	3.55	1.17	0.85	−3.55	0.43	7.36	−3.03
[Cu(L)(phen)] **1c**	−4.90	−2.52	2.38	3.71	1.19	0.84	−3.71	0.42	8.18	−3.11
[Zn(L)(phen)] **1d**	−4.87	−2.67	2.21	3.77	1.10	0.91	−3.77	0.45	7.85	−3.41
[Co(L)(bpy)] **1e**	−5.23	−2.13	3.10	3.68	1.55	0.64	−3.68	0.32	10.52	−2.37
[Ni(L)(bpy)] **1f**	−4.38	−2.01	2.37	3.19	1.19	0.84	−3.19	0.42	6.05	−2.69
[Cu(L)(bpy)] **1g**	−4.92	−2.58	2.34	3.75	1.17	0.85	−3.75	0.43	8.25	−3.20
[Zn(L)(bpy)] **1h**	−4.90	−2.66	2.25	3.78	1.12	0.89	−3.78	0.45	8.02	−3.37

**Table 10:** Molecular docking parameters of complex **1(a–h)** with TS receptor.

Complexes	Docking Score kcal.mol^−1^	Active Sites with a Mode of Interaction		
		H-bond	Π–π Stacking	Hydrophobic Interactions (Cutoff at 5Å)
[Co(L)(phen)] **1a**	−5.700	--	PHE 225	PHE 80, PHE 91, ILE 108, TRP 109, TYR 135, LEU 192, CYS 195, LEU 221, VAL 223, PRO 224, PHE 225, TYR 258
[Ni(L)(phen)] **1b**	−5.615	--	PHE 225	PHE 80, PHE 91, ILE 108, TRP 109, TYR 135, LEU 192, CYS 195, LEU 221, VAL 223, PRO 224, PHE 225, TYR 258
[Cu(L)(phen)] **1c**	−5.791	ASN 226	PHE 225	PHE 80, PHE 91, ILE 108, TRP 109, TYR 135, LEU 192, PRO 193, PRO 194, CYS 195, VAL 223, PHE 225, VAL 238, TYR 258
[Zn(L)(phen)] **1d**	−5.367	ASN 226	--	PHE 80, PHE 91, ILE 108, TRP 109, TYR 135, LEU 192, CYS 195, LEU 221, VAL 223, PHE 225, TYR 258
[Co(L)(bpy)] **1e**	−5.49	ASN 226	--	PHE 80, TRP 90, LEU 101, ALA 111 ILE 108, TRP 109, ALA 111, TYR 135, LEU 192, CYS 195, LEU 221, VAL 223, PHE 225
[Ni(L)(bpy)] **1f**	−4.97	--	TRP 109	PHE 80, TRP 90, LEU 101, ALA 111 ILE 108, TRP 109, ALA 111, TYR 135, LEU 192, PRO 193, PRO 194, CYS 195, LEU 221, VAL 223, PHE 225
[Cu(L)(bpy)] **1g**	−5.026	--	PHE 225	PHE 80, PHE 91, ILE 108, TRP 109, ALA 111, TYR 135, LEU 192, CYS 195, LEU 221, VAL 223, PRO 224, PHE 225, TYR 258
[Zn(L)(bpy)] **1h**	−5.223	ASN 226	--	PHE 80, PHE 91, ILE 108, TRP 109, TYR 135, LEU 192, CYS 195, LEU 221, VAL 223, PHE 225, TYR 258

**Table 11:** Antibacterial activity of metal (II) complexes **1**(**a**–**h**) against pathogenic bacteria tested at various concentrations by an agar diffusion method.

Complexes	Inhibition Zone Measured (mm)											
	*Escherichia coli*				*Klebsiella pneumonia*				*Staphylococcus aureus*			
	Concentration (µL/mL)											
	5	10	15	20	5	10	15	20	5	10	15	20
[Co(L)(phen)] **1a**	7.5 ± 0.6	8.7 ± 0.4	9.8 ± 0.3	10.6 ± 0.4	--	--	7.6 ± 0.5	8.7 ± 0.1	--	7.6 ± 0.5	8.5 ± 0.3	9.3 ± 0.4
[Ni(L)(phen)] **1b**	8.0 ± 0.4	9.1 ± 1.2	10.9 ± 0.4	13.6 ± 0.6	8.3 ± 0.7	9.6 ± 0.6	10.8 ± 1.2	13.9 ± 0.8	7.2 ± 0.2	9.0 ± 0.2	10.6 ± 0.6	12.3 ± 0.4
[Cu(L)(phen)] **1c**	9.8 ± 0.4	12.1 ± 0.2	13.6 ± 0.4	14.6 ± 0.7	8.9 ± 0.8	10.3 ± 0.7	11.8 ± 0.1	12.6 ± 0.3	10.6 ± 0.9	12.8 ± 0.6	14.5 ± 0.9	15.8 ± 0.2
[Zn(L)(phen)] **1d**	7.8 ± 0.6	8.8 ± 0.5	10.2 ± 0.6	12.4 ± 0.5	8.2 ± 0.6	9.7 ± 1.3	10.8 ± 1.1	12.8 ± 0.8	7.6 ± 0.5	8.8 ± 0.7	10.2 ± 0.8	11.8 ± 0.6
[Co(L)(bpy)] **1e**	7.2 ± 0.6	8.3 ± 0.4	9.3 ± 0.3	10.2 ± 0.4	--	--	9.6 ± 0.5	10.7 ± 0.1	--	--	8.3 ± 0.3	9.1 ± 0.4
[Ni(L)(bpy)] **1f**	7.7 ± 0.4	8.4 ± 1.2	9.5 ± 0.6	12.2 ± 0.4	8.8 ± 0.6	10.2 ± 0.8	12.7 ± 1.3	14.7 ± 0.8	7.4 ± 0.4	9.3 ± 0.6	10.9 ± 0.6	12.8 ± 0.4
[Cu(L)(bpy)] **1g**	9.5 ± 1.2	11.6 ± 0.5	12.8 ± 0.9	13.6 ± 0.4	9.2 ± 0.1	10.9 ± 0.6	12.2 ± 0.8	13.8 ± 0.4	10.8 ± 0.7	13.3 ± 0.8	14.5 ± 0.8	15.6 ± 0.9
[Zn(L)(bpy)] **1h**	7.4 ± 0.6	8.6 ± 0.5	9.6 ± 0.6	11.4 ± 0.5	8.8 ± 0.4	10.2 ± 1.1	11.8 ± 1.2	13.7 ± 0.8	7.9 ± 0.5	9.2 ± 0.4	10.8 ± 0.8	13.2 ± 0.6

**Table 12:** In vitro anticancer activity of metal(II) complexes **1c** and **1g** against A549, HeLa, and MCF-7 cancer lines.

S. No	Complexes	Cell lines Tested			
		IC_50_ (μM) *			
		A549	HeLa	MCF-7	NHDF
1.	[Cu(L)(phen)] **1c**	25.95 ± 1.82	26.26 ± 1.06	17.13 ± 0.74	86.46
2.	[Cu(L)(bpy)] **1g**	39.18 ± 0.43	43.14 ± 0.65	33.18 ± 1.14	71.73
3.	cisplatin	17.91 ± 0.12	16.13 ± 0.16	13.01 ± 0.44	94.12

*: is the concentration of a drug needed to inhibit MCF-7 cells by 50%.

## Data Availability

Not applicable.

## Supplementary Materials

The following supporting information can be downloaded at: https://www.mdpi.com/article/10.3390/molecules28072931/s1, Figure S1: FT-IR spectra of metal(II) complexes **1a**–**1h**; Figure S2: ESI-Mass spectra of metal(II) complexes [Co(L)(phen)] **1a** and [Ni(L)(phen)] **1b**; Figure S3: Optimized molecular structure of (i) cobalt(II) complex **1a**, (ii) nickel(II) complex **1b**, (iii) copper(II) complex **1c** and (iv) zinc(II) complex **1d** using B3LYP/LACVP++ basis set; Figure S4: Optimized molecular structure of (v) cobalt(II) complex **1e**, (vi) nickel(II) complex **1f**, (vii) copper(II) complex **1g** and (viii) zinc(II) complex **1h** using B3LYP/LACVP++ basis set.

## Abbreviations

bpy	2,2′-Bipyridyl
DMF	N,N-Dimethylformamide
DPPH	2,2′-Diphenyl-1-picrylhydrazyl
DMEM	Dulbecco’s Modified Eagle’s Medium
DFT	Density functional theory
EPR	Electron paramagnetic resonance spectroscopy
ESI-MS	Electrospray ionization mass spectroscopy
FBS	Fetal bovine serum
FMO	Frontier molecular orbital
HOMO	Highest occupied molecular orbital
IC50	50% of inhibitory concentration
LUMO	Lowest unoccupied molecular orbital
MTT	3-(4,5-Dimethyl-2-thiazolyl)-2,5-diphenyltetrazolium bromide
NHDF	Nontumorigenic human dermal fibroblasts
OPLS	Optimized potentials for liquid simulations
PDB	Protein data bank
phen	1,10-Phenanthroline
RMSD	Root mean square deviation
TBAP	Tetra(n-butyl)ammonium perchlorate
UV–Vis	Ultraviolet–visible
XRD	X-ray diffraction
MCF-7	Michigan Cancer Foundation
HeLa	Henrietta Lacks
