# Supplementary material for: Catalytic, Theoretical, and Biological Investigations of Ternary Metal (II) Complexes Derived from L-Valine-Based Schiff Bases and Heterocyclic Bases
Source: Molecules. 2023 Mar 24;28(7):2931. doi: 10.3390/molecules28072931 (PMC10095770; doi:10.3390/molecules28072931)
Supplement: Supplementary file 1 [file molecules-28-02931-s001.zip › molecules-2204881-SI.pdf]

molecules

Supplementary Materials

Catalytic, Theoretical, and Biological Investigations of Ternary Metal (II) Complexes Derived from L-Valine-Based Schiff Bases and Heterocyclic Bases

Gopalakrishnan Sasikumar [1], Annadurai Subramani [2], Ramalingam Tamilarasan [3], Punniyamurthy Rajesh [4], Ponnusamy Sasikumar [5,*], Salim Albukhaty [6], Mustafa K. A. Mohammed [7], Subramani Karthikeyan [8], Zaidon T. Al-aqbi [9], Faris A. J. Al-Doghachi [10] and Yun Hin Taufiq-Yap [11,12,*]

1 Department of Chemistry, St. Joseph's College of Engineering, Chennai 600 119, Tamil Nadu, India
2 Department of biochemistry, Dwaraka Doss Goverdhan Doss Vaishnav College, Chennai 600 106, Tamil Nadu, India
3 Department of Chemistry, Vel Tech Multi Tech Dr. Rangarajan Dr. Sakunthala Engineering College, Chennai 600 062, Tamil Nadu, India
4 Department of Physics, Vels Institute of Science, Technology and Advance Studies of Basic Science, Chennai 600 017, Tamil Nadu, India
5 Department of Physics, Saveetha School of Engineering, SIMATS, Chennai 602 701, Tamil Nadu, India
6 Department of Chemistry, College of Science, University of Misan, Maysan 62001, Iraq
7 Radiological Techniques Department, Al-Mustaqbal University College, Hillah 51001, Babylon, Iraq
8 Department of Physics, Periyar University Centre for Post Graduate and Research Studies, Dharmapuri 636 701, Tamil Nadu, India
9 College of Agriculture, University of Misan, Al-Amara, Misan 62001, Iraq
10 Department of Chemistry, Faculty of Science, University of Basrah, Basrah 61004, Iraq
11 Catalysis Science and Technology Research Centre, Faculty of Science, Universiti Putra Malaysia, Serdang 43400, Selangor, Malaysia
12 Faculty of Science and Natural Resources, University Malaysia Sabah, Kota Kinabalu 88400, Sabah, Malaysia
* Correspondence: sasijanaki123@gmail.com (P.S.); taufiq@upm.edu.my (Y.H.T.-Y.)

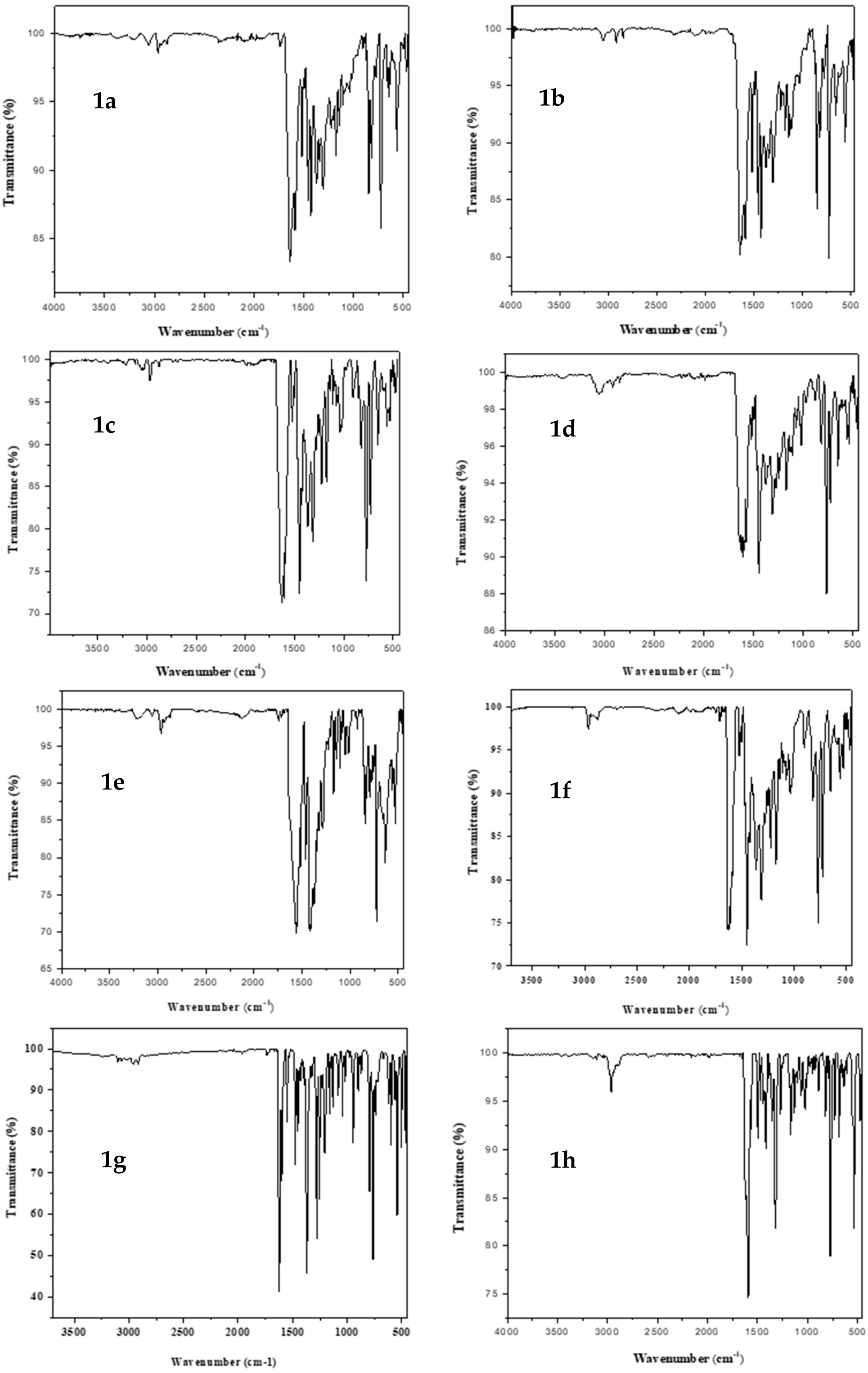

(g) **(h)**

Figure S1. FT-IR spectra of metal(II) complexes **1a–1h.**

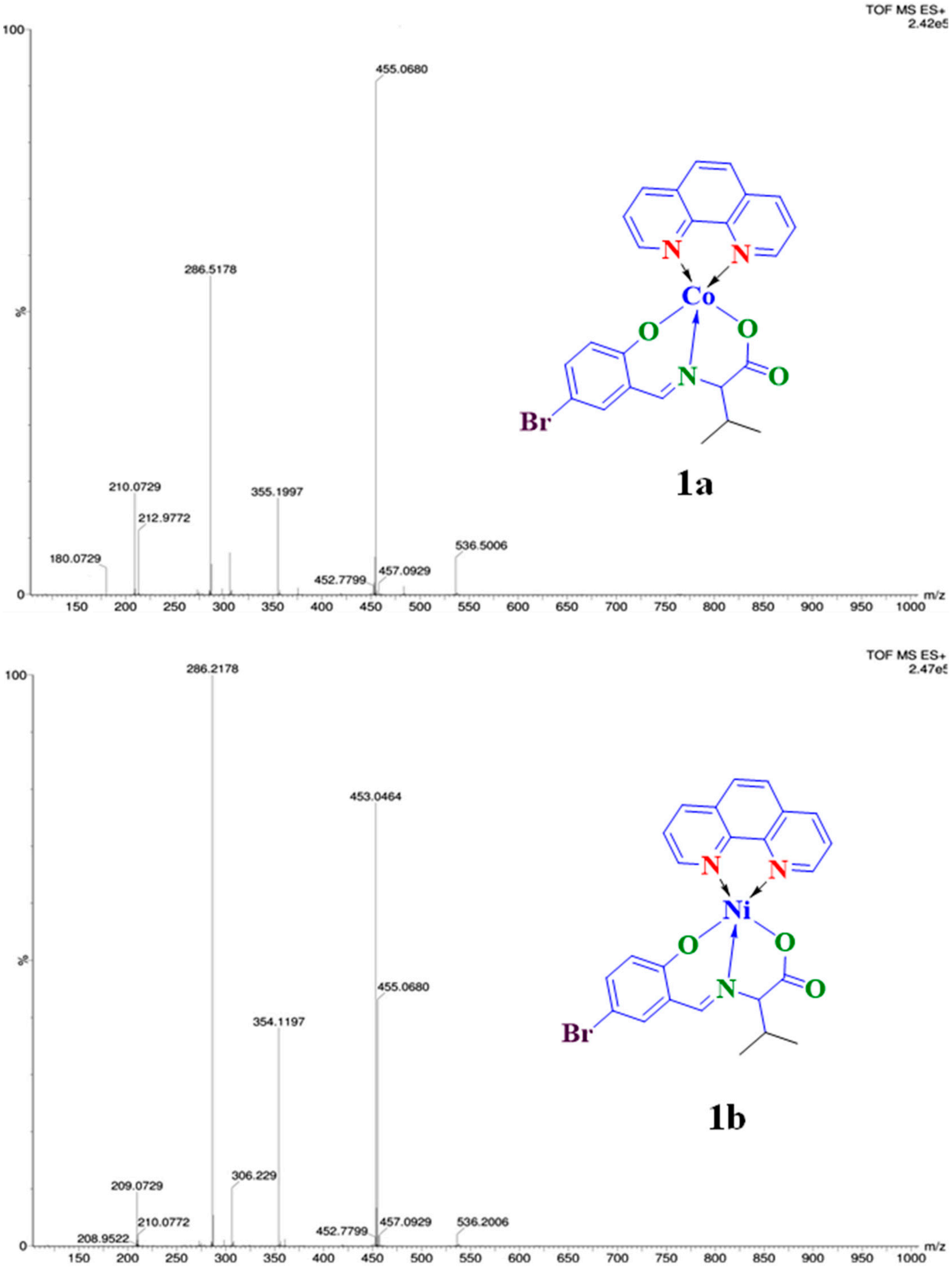

Figure S2. ESI-Mass spectra of metal(II) complexes [Co(L)(phen)] **1a** and[Ni(L)(phen)] **1b.**

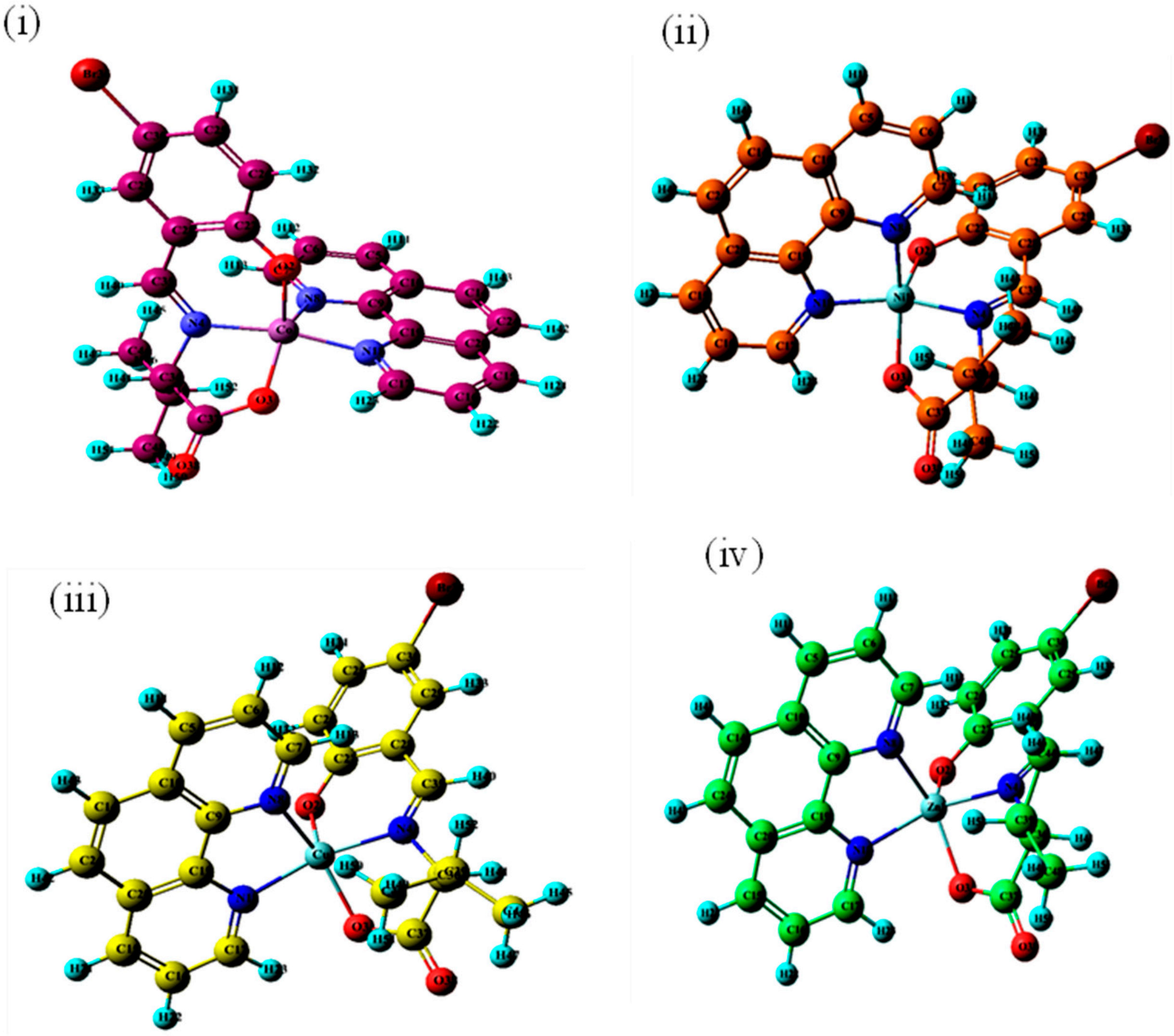

Figure S3. Optimized molecular structure of (i) cobalt(II) complex 1a, (ii) nickel(II) complex 1b, (iii) copper(II) complex 1c and (iv) zinc(II) complex 1d using B3LYP/LACVP++ basis set.

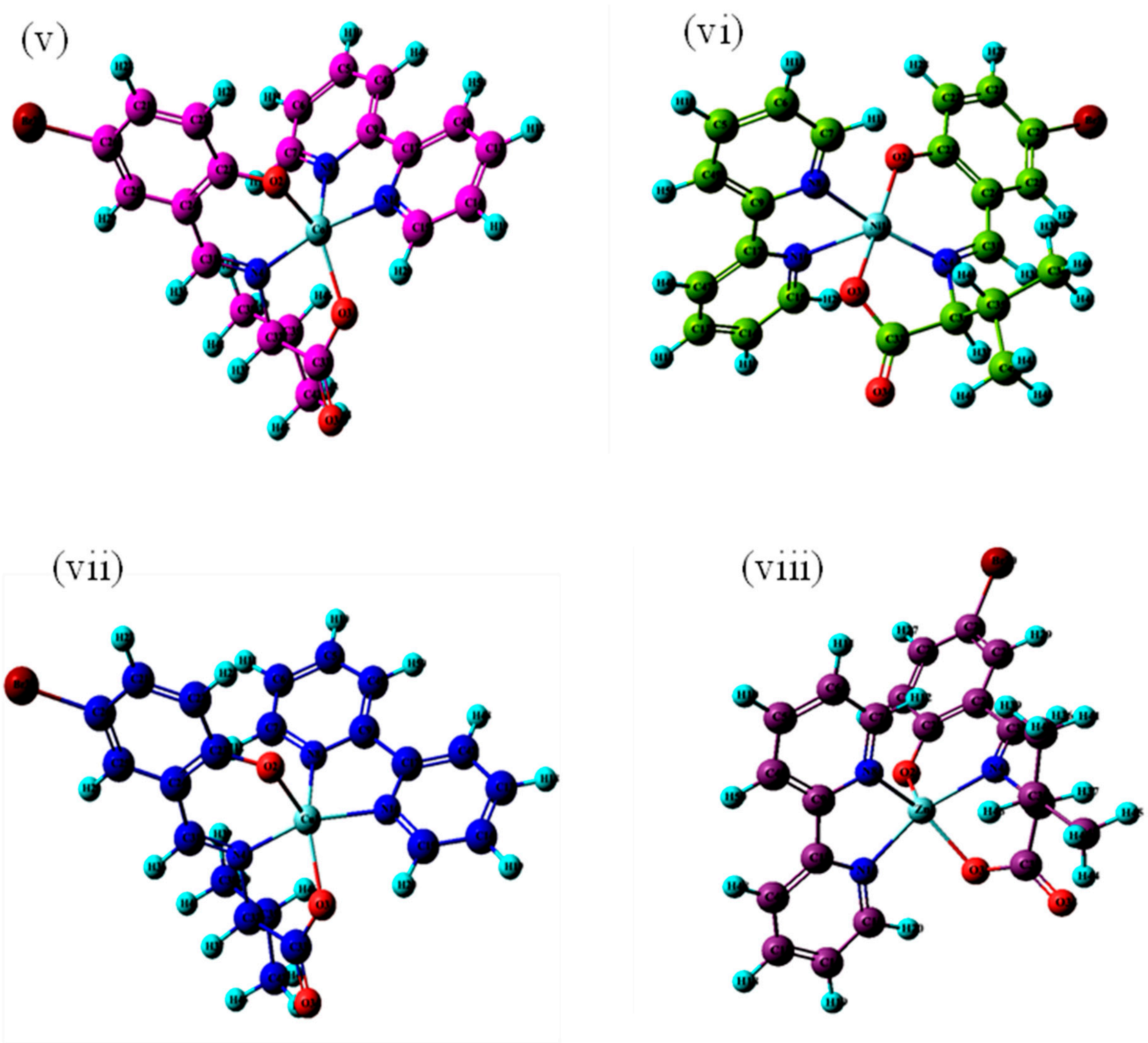

Figure S4. Optimized molecular structure of (v) cobalt(II) complex 1e, (vi) nickel(II) complex 1f, (vii) copper(II) complex 1g and (viii) zinc(II) complex 1h using B3LYP/LACVP++ basis set.
